# Enhancing Aseptic Inflammation Resolution with 1-(2-Ethoxyethyl)-4-(pent-1-yn-1-yl)piperidin-4-yl Propionate: A Novel β-Cyclodextrin Complex as a Therapeutic Agent

**DOI:** 10.3390/molecules29215135

**Published:** 2024-10-30

**Authors:** Symbat Zhumakova, Aliya Tokusheva, Tolganay Zharkynbek, Marina Balabekova, Sulev Koks, Tulegen Seilkhanov, Valery Dembitsky, Alexey Zazybin, Murat Aydemir, Ulan Kemelbekov, Gulgul Kairanbayeva, Valentina Yu

**Affiliations:** 1 Laboratory of Synthetic and Natural Medicinal Compounds Chemistry, A.B. Bekturov Institute of Chemical Sciences, 106 Sh. Ualikhanov St., Almaty 050010, Kazakhstan; symba_t@mail.ru (S.Z.); u.kemelbekov@skma.kz (U.K.); yuvkconst@gmail.com (V.Y.); 2 School of Chemical Engineering, Kazakh-British Technical University, 59 Tole bi St., Almaty 050000, Kazakhstan; azazybin@yahoo.com; 3 Department of Pathological Physiology, Asfendiyarov Kazakh National Medical University, 94 Tole-bi St., Almaty 050000, Kazakhstan; aliyatokusheva88@gmail.com (A.T.); balabekova.m@kaznmu.kz (M.B.); kairanbayeva.g@kaznmu.kz (G.K.); 4 Health Futures Institute, Murdoch University, 90 South St., Perth, WA 6150, Australia; sulev.koks@murdoch.edu.au; 5 Laboratory of Engineering Profile of NMR Spectroscopy, Sh. Ualikhanov Kokshetau State University, 76 Abai St., Kokshetau 020000, Kazakhstan; tseilkhanov@mail.ru; 6 Centre for Applied Research, Innovation and Entrepreneurship, Lethbridge College, 3000 College Drive South, Lethbridge, AB T1K 1L6, Canada; devalery@gmail.com; 7 Department of Chemistry, Faculty of Science, Dicle University, Diyarbakir 21280, Turkey; aydemir@dicle.edu.tr; 8 South Kazakhstan Medical Academy, 1 Al-Farabi Square, Shymkent 160019, Kazakhstan

**Keywords:** 1-(2-ethoxyethyl)-4-(pent-1-yn-1-yl)piperidin-4-yl propionate (***EPPP***), complex ***EPPP*** with β-cyclodextrin (***EPPPβCD***), supracomplex, aseptic inflammation, heavy metal technology-related exposure, ammonium vanadate (NH_4_VO_3_), potassium bichromate (K_2_Cr_2_O_7_)

## Abstract

The synthesized compound, 1-(2-ethoxyethyl)-4-(pent-1-yn-1-yl)piperidin-4-yl propionate (***EPPP***), and its 1:1 complex with β-cyclodextrin (***EPPPβCD***) have been characterized for the first time through a comprehensive suite of analytical methods. This study explores the therapeutic potential of ***EPPPβCD*** in modulating immune responses and accelerating the resolution of septic inflammation induced by chromium and vanadium ions in outbred male rats. The research highlights the significant impact of ***EPPPβCD*** on the dynamics of regulatory T lymphocytes (Tregs), notably causing a reduction in the CD4^+^CD25^+^ fractions at the onset of inflammation. This effect is attributed to the inhibition of Treg proliferation, which is crucial in hastening the resolution of inflammation. These findings underscore the potential of ***EPPPβCD*** as a promising therapeutic agent in controlling and mitigating inflammation mediated by heavy metal exposure, thereby offering a new avenue for the development of anti-inflammatory treatments.

## 1. Introduction

Stable zones of environmental disaster and natural–technological provinces with high concentrations of heavy metals are created by human activity. Research into the toxic effects of ecotoxicants on organisms is extensive [1,2,3,4,5]. Persistent exposure to metals may disrupt immune homeostasis, potentially leading to immunodeficiency, particularly in areas of industrial pollution [6]. Metals like lead, cadmium, chromium, and vanadium can accumulate in body tissues, triggering inflammatory responses. Their toxicity mechanisms include heightened oxidative stress, compromised immune responses, and the activation of inflammatory mediators, resulting in chronic inflammation which may lead to various health issues including respiratory and immune disorders, and potentially cancer [7]. Addressing these adverse effects is crucial for health maintenance.

In vitro studies indicate that air pollution may weaken both innate and adaptive immunity, heightening vulnerability to infections in human and animal models following both short- and long-term exposure [8]. Heavy metals interfere with cellular functions such as growth, proliferation, differentiation, damage repair, and apoptosis, all vital to immune system operations [2]. Disruption of these processes results in significant alterations to the inflammatory response. The prevalent uncertainty surrounding the pathogenesis of inflammatory syndrome hampers the development of effective treatments. Consequently, we are exploring the pathophysiological mechanisms behind inflammation to discover new pathogenetic treatments for poisoning from lead and cadmium salts.

New azacyclic derivatives as potential drugs are of great interest in Medicinal Chemistry. The uniqueness of the chemical structure and the ability to be modified by various molecular fragments allow piperidine derivatives to be widely used to develop compounds with a wide range of pharmacological activity [9]. It was previously discovered that esters of 1-alkoxyalkyl-4-hydroxypiperidines, as their salts with hydrogen chloride, caused local anesthesia, and a pattern of influence of the nature of the acyl residue was revealed, while the most active local anesthetics were benzoic esters [10]. On the other hand, the potential of the 1-(2-ethoxyethyl)piperidine fragment as a myelostimulator is shown [11]. Therefore, a synthetic search for effective pharmacologicals in the N-alkoxyalkylpiperidine acetylenes family seems promising.

In this report, we utilize an aseptic inflammation model to investigate immunopathological changes in animals subjected to two weeks of low-dose exposure to chromium and vanadium. Crucially, we aim to demonstrate the effectiveness of using a complex of 1-(2-ethoxyethyl)-4-(pent-1-yn-1-yl)piperidin-4-yl propionate with cyclodextrin (***EPPPβCD***) for the pathogenetic immunocorrection in animals poisoned with chromium and vanadium salts.

## 2. Results and Discussion

The acetylene derivatives of piperidine exhibit a remarkably broad range of biological effects [12], which has been the principal reason for selecting them as potential immunomodulators.

**Scheme 1:**
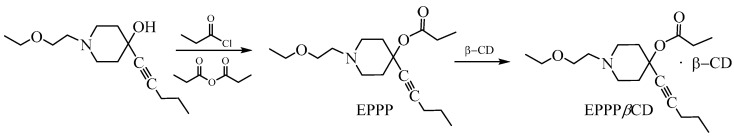
Synthesis of 1-(2-ethoxyethyl)-4-(pent-1-yn-1-yl)piperidin-4-yl propionate and its complex.

Specifically, propylacetylene alcohol of 1-(2-ethoxyethyl)-4-oxopiperidine has been synthesized as a precursor for local anesthetic formulations. This compound was then treated with a mixture of propionyl chloride and propionic anhydride to produce 1-(2-ethoxyethyl)-4-(pent-1-yn-1-yl)piperidin-4-yl propionate (EPPP), achieving a yield of 82.7% (Scheme 1).

The IR spectrum of ***EPPP*** (Figure S1), recorded using KBr pellets, showed characteristic absorptions at the following: 1274.00 cm^−1^ for the C–O stretch, 1744.47 cm^−1^ for the C=O stretch, and 2248.10 cm^−1^ for the C≡C triple bond.

**Figure 1:**
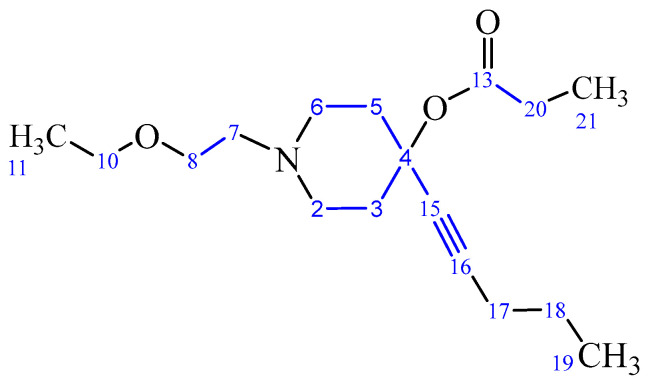
Numbered chemical structure of 1-(2-ethoxyethyl)-4-(pent-1-yn-1-yl)piperidin-4-yl propionate (***EPPP***).

Detailed ^1^H and ^13^C NMR spectra of ***EPPP*** are provided in the following Supplementary Materials: Figures S5 and S6. The chemical shift values of the ^1^H and ^13^C nuclei of ***EPPP*** and β-cyclodextrin in the free state and as part of the complex (***EPPPβCD***) are shown in Table 1 based on numbered chemical structure of ***EPPP*** (Figure 1).

**Figure 2:**
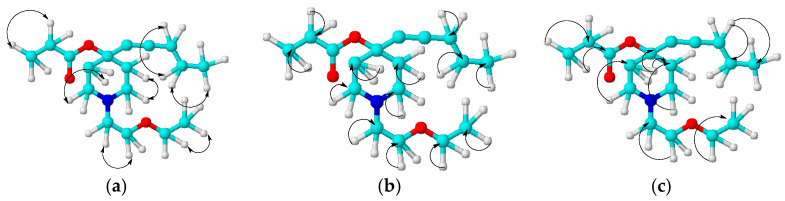
The correlation scheme in the COSY (**a**), HMQC (**b**), and HMBC (**c**) spectra of ***EPPP***.

Additionally, the structure of the propionate is confirmed by two-dimensional NMR techniques including COSY (^1^H-^1^H) (Figure S7), HMQC (^1^H-^13^C) (Figure S8), and HMBC (^1^H-^13^C) (Figure S9), which provide insights into the spin–spin interactions both within and between nuclei, as depicted in the diagrams (Figure 2).

In the ^1^H-^1^H COSY spectra of ***EPPP*** (Figure S7), spin–spin proton correlations are observed across three bonds within the following neighboring methyl–methylene and methylene–methylene groups: H^19^-H^18^ (0.94, 1.44 and 1.44, 0.94), H^21^-H^20^ (1.01, 2.26 and 2.26, 1.01), H^11^-H^10^ (1.09, 3.41 and 3.41, 1.09), and H^7^-H^8^ (2.46, 3.45 and 3.45, 2.46) ppm.

Heteronuclear interactions of protons with carbon atoms through a single bond are established using ^1^H-^13^C HMQC spectroscopy (Figure S8) for the following pairs within the compound: H^21^-C^21^ (1.01, 9.44), H^19^-C^19^ (0.94, 13.63), H^11^-C^11^ (1.09, 15.78), H^18^-C^18^ (1.44, 22.02), H^17^-C^17^ (2.18, 20.41), H^20^-C^20^ (2.26, 28.14), H^3a,5a^-C^3,5^ (1.92, 37.17), H^3e,5e^-C^3,5^ (2.02, 36.85), H^2a,6a^-C^2,6^ (2.40, 50.18), H^2e,6e^-C^2,6^ (2.58, 50.18), H^7^-C^7^ (2.46, 57.48), H^10^-C^10^ (3.41, 66.08), and H^8^-C^8^ (3.45, 68.34) ppm.

The heteronuclear interactions through two or more bonds are established using ^1^H-^13^C HMBC spectroscopy (Figure S9) for pairs such as the following: H^19^-C^18^ (0.94, 21.01), H^21^-C^20^ (1.01, 28.28), H^21^-C^13^ (1.01, 172.55), H^11^-C^10^ (1.09, 66.18), H^18^-C^19^ (1.44, 13.53), H^18^-C^17^ (1.44, 20.74), H^18^-C^16^ (1.44, 87.02), H^17^-C^19^ (2.18, 13.26), H^17^-C^18^ (2.18, 22.35), H^17^-C^15^ (2.18, 80.88), H^17^-C^16^ (2.18, 87.21), H^20^-C^21^ (2.26, 9.78), H^20^-C^13^ (2.26, 172.81), H^2a,6a^-C^8^ (2.40, 68.58), H^10^-C^11^ (3.41, 15.40), H^10^-C^8^ (3.41, 68.31), H^8^-C^7^ (3.45, 57.36), and H^8^-C^10^ (3.45, 66.18) ppm.

The 1-(2-ethoxyethyl)-4-(pent-1-yn-1-yl)piperidin-4-yl propionate is an oily substance, and its inclusion in β-CD facilitates the transformation of the oily product into a stable, solid powder. This modification enhances the compound’s stability in aqueous solutions, reduces oxidation in air, and minimizes dehydration and evaporation. The formation of a β-CD inclusion complex is expected to increase the bioavailability of the propionate compound while simultaneously reducing its toxicity. These factors underscore the potential of β-CD complexation as a strategy to improve the pharmacological profile of piperidine derivatives for therapeutic applications.

The inclusion complex was obtained by mixing ethanol solution of propionate with distilled water solution of β-cyclodextrin in 1:1 ratio. The mixture was placed in a drying oven at 50–55 °C for evaporation. As a result, the complex formed with a yield of 97.2% as a white powder.

The IR and NMR spectroscopy were used for confirmation of complex formation by comparing the spectral data of the initial reagents (***EPPP*** and β-CD) with that of the final product. In Figure S2, the comparative IR spectra of *β*-cyclodextrin and ***EPPPβCD*** is shown; it turned out that the ***EPPPβCD*** spectrum is very similar to *β*-cyclodextrin. In contrast, the proton NMR data provided compelling evidence for the formation of the target complex (***EPPPβCD***).

The most significant changes in chemical shifts within the proton spectrum of β-CD were observed for the H-3 protons directed into the cyclodextrin cavity (∆δ = −0.04 ppm). The cyclodextrin proton H-5 showed a change in chemical shift of −0.02 ppm during complex formation. The other cyclodextrin protons showed the same change in chemical shift of 0.02 ppm during interaction with ***EPPP***. These observations suggest the formation of mixed ***EPPPβCD*** supracomplexes.

During the supramolecular self-assembly of ***EPPP*** molecules with cyclodextrin oligomers, the methylene protons H-8,8, H-7,7, and H-10,10 of the 4-(pentyn-1-yl) and 1-(2-ethoxyethyl) fragments exhibited significant changes in chemical shifts (∆δ = −0.05–0.07 ppm) and deshielding, similarly affecting the H-2e,6e protons of the piperidine ring. The methylene protons H-20,20 and H-17,17 of these fragments showed slight changes in chemical shifts (∆δ = ±0.01 ppm). All protons of the 1-(2-ethoxyethyl) group experienced the same screening or deshielding effect (∆δ = ±0.01 ppm). It is suggested that protons undergoing the most significant chemical shift changes are involved in supramolecular interactions with the glucopyranose protons of β-CD during the self-assembly of complexes.

A comparison of the integral intensities between the protons of β-CD and the ***EPPP*** molecule indicates that there is approximately one molecule of β-CD for each propionate molecule in the ***EPPPβCD*** supracomplex.

**Figure 3:**
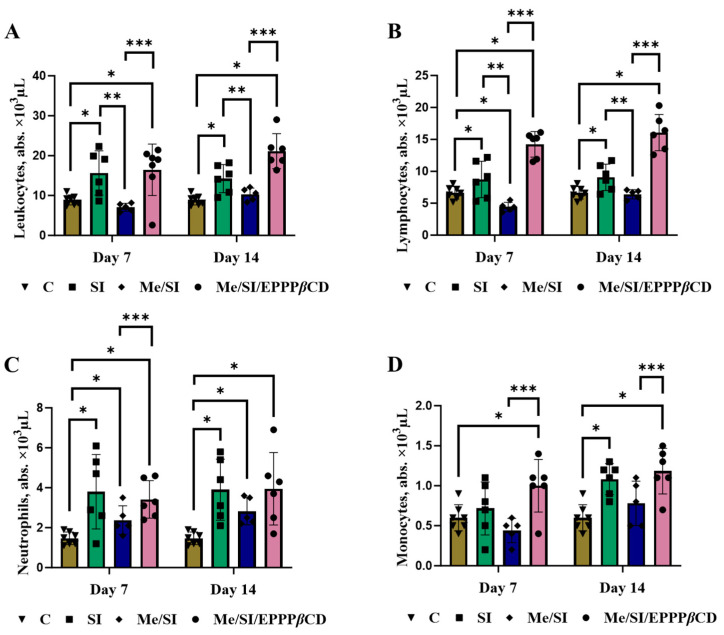
The content of leukocytes in the blood of the experimental rats was monitored over time (hematological blood tests, showing absolute values, were conducted for the animals in the Control, SI, Me/SI, and Me/SI/***EPPPβCD*** groups on both the 7th and 14th days). The results are categorized into the following: (**A**)—Leukocytes; (**B**)—Lymphocytes; (**C**)—Neutrophils; (**D**)—Monocytes. The analysis included assessing significant differences between the groups using the Student’s *t*-test, with significance levels set at * *p* < 0.05, ** *p* < 0.01, *** *p* < 0.001.

To explore the biological activity of the ***EPPP*** complex with beta-cyclodextrin, its impact on the blood components of rats was studied. Peripheral blood is known to quickly reflect the earliest changes in the body, with leukocytes being primary responders to external stimuli, influencing the course and outcome of inflammation. This study concurrently analyzed the blood of rats exposed to both vanadium and chromium intoxication and to turpentine effects. Hematological results, after treating rats with ammonium vanadate and potassium dichromate salts for two weeks, are detailed in Figure 3.

Inflammatory processes typically trigger the activation of the body’s immunological defenses, primarily through blood cellular components. Notably, after 7 days, the SI group (not previously defined in the text) showed a significant increase in total leukocyte count by 69.7% compared to the control, driven by rises in neutrophils and lymphocytes by 153.3% and 59.1%, respectively. These levels persisted above control values after 14 days.

Conversely, the Me/SI group displayed a unique blood profile with leukocyte levels maintained at control values. However, the absolute lymphocyte count after 7 days was 58.1% lower than in the SI group (*p* = 0.0076) and remained 29.7% lower after 14 days (*p* = 0.0245).

In the comparison of the neutrophil fractions in the blood for the SI and Me/SI groups, it was observed that the neutrophil levels in the Me/SI group, though statistically higher than the control by 60% and 86.7%, respectively, did not reach the levels observed in the SI group.

The decrease in monocytes observed on the 7th day in the Me/SI group reverted to control levels by the 14th day. However, the SI group showed a significant increase in monocytes of 83.3% compared to the control on the 14th day (*p* = 0.0019). This increase suggests that, in the context of aseptic inflammation, with prior exposure to vanadium and chromium salts, there was no increase in the lymphocyte fraction in the Me/SI group. Thus, it was concluded that vanadium and chromium adversely affect blood lymphocytes.

Prior to the study, the affected animals had been treated with ***EPPPβCD*** for two weeks. After 7 days, the following notable increases were observed in the Me/SI/***EPPPβCD*** group: leukocyte levels were 2.1 and 2.6 times higher than the Control and Me/SI groups, lymphocyte counts were 2.1 and 3.2 times higher, and monocyte counts were 1.7 and 2.5 times higher, respectively. Neutrophil levels exceeded both the control and previous period levels by more than 1.5 times.

**Figure 4:**
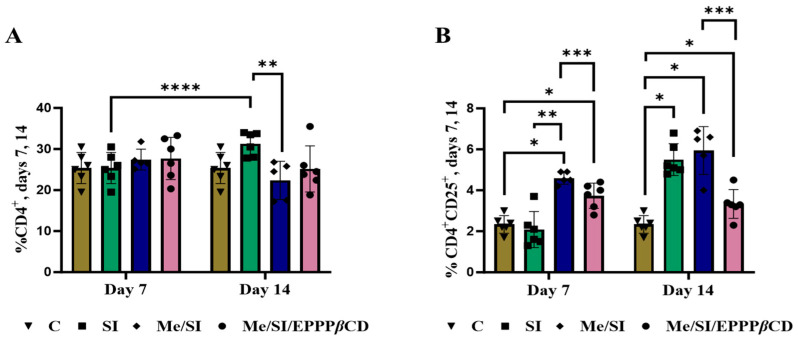
The proportion of CD4^+^ and CD4^+^CD25^+^ lymphocytes was analyzed in animals with aseptic inflammation treated with ***EPPPβCD*** (immunophenotyping of spleen cells from the Control, SI, Me/SI, and Me/SI/***EPPPβCD*** groups was conducted on the 7th and 14th days. The results are categorized into the following: (**A**)—%CD4^+^ lymphocytes; (**B**)—%CD4^+^CD25^+^ lymphocytes. Significant differences between the groups were determined using the Student’s *t*-test, with significance thresholds set at * *p* < 0.05, ** *p* < 0.01, *** *p* < 0.001, **** *p* < 0.0001.

Furthermore, the regulation of inflammation involves T-reg cells. The results from the studies on the subcellular populations in the spleens of experimental animals, focusing on phenotypes of effector and regulatory lymphocytes and the effect of ***EPPPβCD*** on their proliferative activity, are detailed in Figure 4.

The proportion of splenocytes with the Treg cell phenotype (CD4^+^CD25^+^) showed no change in the SI group within the first 7 days of aseptic inflammation onset. However, by the 14th day, a more than two-fold increase in these cells was observed (M = 5.5, SD = 0.8; p_control = 0.001), compared to the control (M = 2.3, SD = 0.4), as depicted in Figure 4B. In contrast, in the Me/SI group, there was already a 100% increase in the proportion of CD4^+^CD25^+^ cells by the 7th day (M = 4.6, SD = 0.3, p_control < 0.0001), compared to the control level (M = 2.3, SD = 0.4). This upward trend continued to the 14th day, with a 156.5% difference compared to the control (M = 5.9, SD = 1.2; p_control = 0.001).

After 7 days, analysis of surface expression showed that ***EPPPβCD*** influenced the immunophenotype of lymphocytes, reducing CD4^+^CD25^+^ expression compared with the Me/SI group. The expression level was 3.7% (SD = 0.6), which is 19.6% lower than in the Me/SI group (M = 4.6, SD = 0.3; p_Me/SI = 0.017). After 14 days, no statistically significant differences were observed in the relative content of CD4^+^ among T-lymphocytes. However, the differentiation of CD4^+^CD25^+^ cells within the CD4^+^ T-lymphocyte pool, influenced by ***EPPPβCD***, decreased by 2 times compared with the Me/SI group.

In studying the impact of heavy metal salt complexes on key immune defense mechanisms, we observed that vanadium and chromium inhibit the activity of effector T cells, leading to a protracted course of aseptic inflammation. The CD4^+^ T cells are typically categorized into regulatory T cells (Tregs) and conventional T helper cells [13]. Unlike inflammatory (effector) T cells, only Tregs are capable of inhibiting immune responses, and an imbalance between effector T cells and Tregs often results in loss of tolerance and disease progression. The Tregs are instrumental in modulating effector T cell activity through the secretion of inhibitory cytokines such as transforming growth factor TGF-β1 and IL-10 [14]. Ammonium metavanadate and potassium dichromate shift the production from pro-inflammatory IL-6 to the anti-inflammatory IL-10 during the acute phase of inflammation. Treatment with the drug polyoxidonium (azoximer bromide) was shown to prevent damage to neutrophil membranes and significantly enhance neutrophil phagocytic activity in vitro [15], as demonstrated by the nitro blue tetrazolium test in a model of aseptic inflammation induced by vanadium and chromium intoxication. Polyoxidonium stimulates T cell proliferation, promotes dendritic cell expansion and maturation, and increases endosomal hydrogen peroxide concentrations, which in turn activate signaling molecules and transcription factors such as NF-κB with detoxifying and antioxidant effects [16]. It is likely that the mechanism of action of EPPPβCD is analogous to that of polyoxidonium.

## 3. Materials and Methods

### 3.1. Chemical Experimental Part

#### 3.1.1. The Synthesis and Structure Studies for the Chemical Compounds

Reagents used in synthesis were purchased from Merck (Boston, MA, USA) and included Propionic anhydride, ≥99% (CAS No. 123-62-6); Propionyl chloride, 98% (Cas No. 79-03-8). The 1-(2-ethoxyethyl)-4-(pent-1-yn-1-yl)piperidin-4-ol was synthesized, purified and identified according to [17]. The progression of reactions and the purity of the synthesized products were assessed using thin-layer chromatography (TLC) on alumina (Al_2_O_3_) plates. The spots of the substances were visualized with iodine vapor. For TLC, a benzene–ethanol mixture (3:1) was used as the eluent. Elemental analysis was conducted using a Rapid Micro N Cube elemental analyzer to confirm the composition of the products. The infrared (IR) spectra were recorded on a Bruker Alpha-P ATR FTIR (diamond crystal) spectrometer (Bruker, Billerica, MA, USA) using potassium bromide (KBr) pellets, operating range 400–4000 cm^−1^. Additionally, the ^1^H and ^13^C NMR spectra of the samples were recorded using a JNM-ECA 400 (JEOL, Tokyo, Japan) spectrometer, operating at frequencies of 399.78 MHz for ^1^H and 100.53 MHz for ^13^C, in deuterated dimethyl sulfoxide (DMSO-d_6_). The residual signal of solvent DMSO-d_6_ at 2.5 ppm was used as a chemical shift standard. Each measurement was conducted a minimum of three times and reviewed in instances of notable deviations from the mean. The findings are applicable to a broader spectrum of substrates.

#### 3.1.2. The Synthesis of 1-(2-Ethoxyethyl)-4-(pent-1-yn-1-yl)piperidin-4-yl Propionate (EPPP)

To synthesize 1-(2-ethoxyethyl)-4-(pent-1-yn-1-yl)piperidinyl-4 propionate (***EPPP***), a mixture was prepared by adding 1.50 g (0.00063 mol) of 1-(2-ethoxyethyl)-4-(pent-1-yn-1-yl)piperidin-4-ol [17], 8.15 g (0.063 mol) of propionic anhydride, and 5.80 g (0.063 mol) of propionyl chloride. An excess mixture of propionic anhydride and propionyl chloride was also used as solvent for reaction. During the adding of all reagents, the release of heat was observed. After cooling the reaction mixture to room temperature, the mixture was continuously mixed for 3 days. Afterward, the excess reagents were removed under vacuum using a water-jet pump. Water was then added to the resulting oil, which was subsequently treated with potash. The organic phase was extracted with benzene and dried over anhydrous magnesium sulfate (MgSO_4_). The drying agent was filtered off, and the solvent was evaporated to yield 1.53 g (82.7%) of ***EPPP*** as a light-yellow oil.

The elemental composition of ***EPPP***, C_17_H_29_NO_3_, was calculated as follows: Carbon (C) 69.12%, Hydrogen (H) 9.89%, Nitrogen (N) 4.74%. The analytical results found were as follows: Carbon (C) 69.14%, Hydrogen (H) 9.94%, Nitrogen (N) 4.75%.

IR (KBr, ν, cm^−1^): see Figure S1.

^1^H NMR and ^13^C NMR (DMSO-d_6_, δ, ppm): see Table 1 and Figures S6–S9.

#### 3.1.3. The Complex of 1-(2-Ethoxyethyl)-4-(pent-1-yn-1-yl)piperidin-4-yl Propionate with β-Cyclodextrin (EPPPβCD)

To prepare the complex of propionate of 1-(2-ethoxyethyl)-4-(pent-1-yn-1-yl)piperidinyl-4 with β-cyclodextrin, 0.80 g (0.0027 mol) of the propionate in 30 mL of ethyl alcohol was mixed with 3.08 g (0.0027 mol) of β-cyclodextrin dissolved in 50 mL of distilled water. The mixture was placed in a drying oven, where ethanol and water were evaporated at 50–55 °C. This process resulted in 3.77 g (97.2%) of the complex in the form of a white powder, which melts with decomposition above 240 °C.

The elemental analysis for the complex, formulated as C_59_H_99_O_38_N, was calculated to contain Carbon (C) 49.55%, Hydrogen (H) 6.93%, and Nitrogen (N) 0.98%. The values found from the analysis were as follows: Carbon (C) 49.51%, Hydrogen (H) 6.99%, and Nitrogen (N) 0.97%.

IR (KBr, ν, cm^−1^): see Figures S2 and S3.

^1^H NMR and ^13^C NMR (DMSO-d_6_, δ, ppm): see Table 1 and Figures S10 and S19.

For β-cyclodextrin:

IR (KBr, ν, cm^−1^): see Figure S4.

^1^H NMR and ^13^C NMR (DMSO-d_6_, δ, ppm): see Table 1 and Figures S15 and S19.

### 3.2. Biological Experimental Part

*Reagents used.* The experimental protocol involved the use of the following reagents: Phosphate Buffer Saline (PBS, Sigma-Aldrich, Darmstadt, Germany), 0.9% saline solution (Kelun-Kazpharm LLP, Almaty, Kazakhstan), nylon filters with a mesh size of 70 µm (Fisherbrand™, Leicestershire, UK), High-Yield Lyse reagent for erythrocyte lysis (Invitrogen™, Alcobendas, Spain), CytoFix Fixation Buffer for cell fixation (BD Biosciences, San Jose, CA, USA), monoclonal antibodies against CD4 (0.25 µg/sample, clone OX35, PerCP-eFluor™ 710 fluorophore, eBioscience™, Torrance, CA, USA), and monoclonal antibodies against CD25 (0.125 µg/sample, clone OX39, APC fluorophore, eBioscience™, USA).

*Animals used.* The experiments were conducted on outbred male rats, 8–12 months old, weighing 180–220 g, housed under standard vivarium conditions, and fed a standard diet. All animal procedures were approved by the Local Ethics Commission of S.D. Asfendiyarov Kazakh National Medical University (Protocol No. 4 (95), dated 29 April 2020) and complied with both international [18] and national [19] animal welfare regulations. The rats had free access to food and water and were kept in a room with temperatures of 22.5–23.0 °C and relative humidity of 50–70%.

*Experimental design.* The inflammatory process was modeled on the 7th and 14th days to observe significant changes in immune responses. The animals were divided into the following four groups of 20 each:

Group 1 (Control) received no intervention.

Group 2 (SI) had sterile inflammation induced [20,21] via subcutaneous injection of 0.3 mL of turpentine oil, following shaving and disinfection with 70% ethanol.

Groups 3 (Me/SI) and 4 (Me/SI/***EPPPβCD***) were pre-treated with ammonium vanadate and potassium dichromate dissolved in PBS orally for two weeks at a dosage of 5 mg/kg, except Sundays. The rats received 0.43 mg of vanadium and 0.34 mg of chromium daily, accumulating 6.02 mg and 4.76 mg, respectively, over two weeks [22,23,24].

After the metal salt administration, aseptic inflammation was induced, with Group 4 also receiving ***EPPPβCD*** subcutaneously at 50 mg/kg (in saline solution) for 10 days post-turpentine injection. The 50 mg/kg dose demonstrated the optimal balance between efficacy and safety when compared to both the 10 mg/kg and 100 mg/kg doses.

On days 7 and 14 post-premedication, blood was collected under anesthesia, and spleens were removed and processed to assess immunological markers using flow immunocytofluorimetry. Hematological parameters were analyzed using a Sysmex 1000i hematology analyzer.

*Flow cytometry.* Markers were determined by flow immunocytofluorimetry. For this purpose, the cell suspension was treated with monoclonal antibodies to surface markers, according to the manufacturer’s protocols. Nonspecific fluorescence was monitored using FMO controls. The resulting cell preparations were passed through a Thermo Fisher Scientific Attune™, Waltham, MA, USA, NxT flow cytometer and the percentage of labeled cells was assessed.

Sample preparation and staining of splenocytes for flow cytometry. Before isolating cells from half or a third of an organ measuring 0.5 cm × 2 cm, the spleen of rats was placed in 0.5 mL of cold physiological solution (Kelun-Kazpharm LLP, Almaty, Kazakhstan). The 4.5 mL of cold saline was added to the crushed tissue suspension, after filtration through disposable nylon filters with 70 micrometer mesh size (Fisherbrand™, UK), and then centrifugation at 400 G for 5 min. The supernatant was collected as much as possible, without disturbing the sediment, trying not to catch pieces of tissue with the pipette.

For selective lysis of erythrocytes in the washed cell mass obtained by grinding the spleen, we used the ready-made High-Yield Lyse reagent (Invitrogen™, Spain) in a volume of 2 mL, resuspending the precipitated cells and keeping the reagent for 10 min. Then, from the pre-mixed solution with splenocytes, the liquid was carefully taken into clean, labeled microtubes, without touching pieces of tissue, large fragments of suspension, and other undissolved components of the solution. After lysis, the cells were washed twice in 2 mL and, subsequently, the cells were resuspended in 100 μL of chilled physiological solution, after which cell surface markers were stained using monoclonal antibodies to CD4 (0.25 μg/sample, clone OX35, conjugated with the fluorophore PerCP-eFluor™ 710 (eBioscience™) and monoclonal antibodies to CD25 (0.125 μg/sample, clone OX39, APC fluorophore, eBioscience™) for 30 min in the dark when cooled to 4 °C. After staining, the cells were washed in 2 mL of physiological solution, the sediment was fixed and 500 μL of 0.9% physiological solution was added, the sediment was mixed by vortexing or pipetting. The results were analyzed on a flow cytometer.

The Attune™ NxT Acoustic Focusing Cytometer is controlled by the Attune™ NxT Cytometrix Software, version 6.0.1 (Thermo Fisher Scientific).

**Figure 5:**
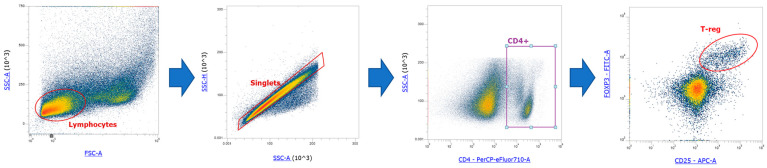
General gating strategy.

*Gating strategy* (Figure 5). For all samples, before choosing a test for the significance of deviations, the sample was checked for normality of distribution using the Smirnov–Kolmogorov test. These actions are required for statistical processing.

*Notes.* Lymphocytes were selected based on light scattering parameters (cell morphology), then co-incident events (unseparated cells) were removed. Single lymphocytes were analyzed for the presence of CD4, and cells positive for this marker were in turn analyzed for the simultaneous presence of CD25 proteins, and were taken for regulatory T cells.

All the experiments were carried out in at least 10-fold repetition. Using the Excel application program, the arithmetic mean (M) and standard deviation (SD) were calculated. Graphs and figures contain information in the form of arithmetic means (M) ± standard deviation (SD). The significance of the difference in mean values between two experiments was calculated using the TTEST program. Differences were considered insignificant if the probability of the null hypothesis did not exceed 5% (*p* > 0.05). Results were graphically represented using GraphPad Prism 10.

## 4. Conclusions

It has been determined that 1-(2-ethoxyethyl)-4-(pent-1-yn-1-yl)piperidin-4-yl propionate (***EPPP***) was synthesized with an 82.7% yield. This formation suggests a significant interaction between the molecules, potentially enhancing the biological availability and stability of EPPP.

The inflammatory process has been shown to activate the body’s immunological defense mechanisms, primarily through the cellular components of the blood. The study revealed that there is no increase in the lymphocytic fraction of the blood, suggesting that vanadium and chromium adversely affect blood lymphocytes.

The toxic effects of chromium and vanadium include the generation of reactive oxygen species, causing oxidative stress, activation of lipid peroxidation, and apoptosis [25].

The administration of ***EPPPβCD*** stimulated the proliferative and phagocytic activities of lymphocytes and monocytes, showing an enhanced effect over the duration of the experiment, which is obviously associated with the restoration of cell membrane permeability, similar to treatment with polyoxidonium.

Furthermore, ***EPPPβCD*** impacted the activity of regulatory T lymphocytes (Tregs), leading to a reduction in the total CD4^+^CD25^+^ fractions at the onset of inflammation. This reduction is presumed to result from the inhibition of Treg production, significantly accelerating the resolution of inflammation.

Overall, ***EPPP*** in its complex form with β-cyclodextrin, ***EPPPβCD***, is recognized as a promising biologically available preparation with a pronounced protective effect against chromium and vanadium poisoning. This suggests its potential use in therapeutic applications aimed at mitigating the adverse effects of these metals.

## Figures and Tables

**Table 1:** The chemical shift values of the ^1^H and ^13^C nuclei of ***EPPP*** and β-cyclodextrin in the free state (δ_0_) and as part of the complex (***EPPPβCD***) (δ).

Atom No.	CH_x_ Group	δ_0_, ppm.		δ, ppm.		∆δ = δ − δ_0_	
		^1^H	^13^C	^1^H	^13^C	^1^H	^13^C
***EPPP***							
2a, 6a	CH_2_	2.40 widened s	50.19	2.38 widened s	50.19	−0.02	0
2e, 6e		2.58 widened s		2.53 widened s		−0.05	
3a, 5a	CH_2_	1.92 widened s	37.01	1.92 widened s	37.02	0	0.01
3e, 5e		2.02 widened s		2.03 widened s		0.01	
4	>C<	-	73.51	-	73.54	−	0.03
7	CH_2_	2.45–2.47 m	57.43	2.39–2.43 m	57.43	−0.05	0
8	CH_2_	3.44–3.45 m	68.31	3.49–3.51 m	68.29	−0.05	−0.02
10	CH_2_	3.40–3.42 m	65.93	3.47–3.49 m	65.93	0.07	0
11	CH_3_	1.08–1.10 m	15.56	1.08–1.10 m	15.62	0	0.06
13	>CO	-	172.26	-	172.32	−	0.06
15	C≡	-	80.48	-	80.50	−	0.02
16	C≡	-	87.20	-	87.25	−	0.05
17	CH_2_	2.17–2.19 m	20.35	2.16–2.18 m	20.35	−0.01	0
18	CH_2_	1.43–1.46 m	22.02	1.45–1.47 m	22.03	0.02	0.01
19	CH_3_	0.93–0.95 m	13.57	0.94–0.96 m	13.57	0.01	0
20	CH_2_	2.25–2.27 m	28.05	2.24–2.26 m	28.06	−0.01	0.01
21	CH_3_	1.00–1.02 m	9.46	0.99–1.01 m	9.51	−0.01	0.05
***EPPPβCD***							
1	>CH	4.82 s	102.42	4.84 m	102.43	0.02	0.01
2	>CH	3.30–3.34 m	72.86	3.33–3.34 m	72.88	0.02	0.02
3	>CH	3.52–3.56 m	73.54	3.49–3.51 m	73.54	−0.04	0
4	>CH	3.32–3.36 m	82.01	3.37–3.40 m	82.03	0.02	0.02
5	>CH	3.52 s	72.51	3.49–3.51 m	72.52	−0.02	0.01
6	CH_2_	3.61–3.62 m	60.42	3.63–3.65 m	60.41	0.02	−0.01

## Data Availability

The datasets used and/or analyzed during the present study are available from the corresponding author on reasonable request.

## Supplementary Materials

The following supporting information can be downloaded at: https://www.mdpi.com/article/10.3390/molecules29215135/s1, Figure S1: IR (KBr, ν, cm^−1^) spectrum of 1-(2-ethoxyethyl)-4-(pent-1-yn-1-yl)piperidinyl-4 propionate (***EPPP***); Figure S2 IR (KBr, ν, cm^−1^) spectrum of β-cyclodextrin (β-CD) and the complex of 1-(2-ethoxyethyl)-4-(pent-1-yn-1-yl)piperidin-4-yl propionate with β-cyclodextrin (***EPPPβCD***); Figure S3: IR (KBr, ν, cm^−1^) spectrum of the complex of 1-(2-ethoxyethyl)-4-(pent-1-yn-1-yl)piperidin-4-yl propionate with β-cyclodextrin (***EPPPβCD***); Figure S4: IR (KBr, ν, cm^−1^) spectrum of β-cyclodextrin (β-CD); Figure S5: ^1^H NMR (399.78 MHz, DMSO-d_6_) spectrum of 1-(2-ethoxyethyl)-4-(pent-1-yn-1-yl)piperidinyl-4 propionate (***EPPP***); Figure S6: ^13^C NMR (100.53 MHz, DMSO-d_6_) spectrum of 1-(2-ethoxyethyl)-4-(pent-1-yn-1-yl)piperidinyl-4 propionate (***EPPP***); Figure S7: ^1^H-^1^H COSY spectrum of 1-(2-ethoxyethyl)-4-(pent-1-yn-1-yl)piperidinyl-4 propionate (***EPPP***); Figure S8: ^1^H-^13^C HMQC spectrum of 1-(2-ethoxyethyl)-4-(pent-1-yn-1-yl)piperidinyl-4 propionate (***EPPP***); Figure S9: ^1^H-^13^C HMBC spectrum of 1-(2-ethoxyethyl)-4-(pent-1-yn-1-yl)piperidinyl-4 propionate (***EPPP***); Figure S10: ^1^H NMR (399.78 MHz, DMSO-d_6_) spectrum of the complex of 1-(2-ethoxyethyl)-4-(pent-1-yn-1-yl)piperidin-4-yl propionate with β-cyclodextrin (***EPPPβCD***); Figure S11: ^13^C NMR (100.53 MHz, DMSO-d_6_) spectrum of the complex of 1-(2-ethoxyethyl)-4-(pent-1-yn-1-yl)piperidin-4-yl propionate with β-cyclodextrin (***EPPPβCD***); Figure S12: ^1^H-^1^H COSY spectrum of the complex of 1-(2-ethoxyethyl)-4-(pent-1-yn-1-yl)piperidin-4-yl propionate with β-cyclodextrin (***EPPPβCD***); Figure S13: ^1^H-^13^C HMQC spectrum of the complex of 1-(2-ethoxyethyl)-4-(pent-1-yn-1-yl)piperidin-4-yl propionate with β-cyclodextrin (***EPPPβCD***); Figure S14: ^1^H-^13^C HMBC spectrum of the complex of 1-(2-ethoxyethyl)-4-(pent-1-yn-1-yl)piperidin-4-yl propionate with β-cyclodextrin (***EPPPβCD***); Figure S15: ^1^H NMR (399.78 MHz, DMSO-d_6_) spectrum of β-cyclodextrin (β-CD); Figure S16: ^13^C NMR (100.53 MHz, DMSO-d_6_) spectrum of β-cyclodextrin (β-CD); Figure S17: ^1^H-^1^H COSY spectrum of β-cyclodextrin (β-CD); Figure S18: ^1^H-^13^C HMQC spectrum of β-cyclodextrin (β-CD); Figure S19: ^1^H-^13^C HMBC spectrum of β-cyclodextrin (β-CD);.
