# Supplementary material for: Enhancing Aseptic Inflammation Resolution with 1-(2-Ethoxyethyl)-4-(pent-1-yn-1-yl)piperidin-4-yl Propionate: A Novel β-Cyclodextrin Complex as a Therapeutic Agent
Source: Molecules. 2024 Oct 30;29(21):5135. doi: 10.3390/molecules29215135 (PMC11547506; doi:10.3390/molecules29215135)
Supplement: Supplementary file 1 [file molecules-29-05135-s001.zip › molecules-3277909-supplementary.pdf]

Supporting information

The IR spectra were recorded on a Bruker Alpha-P ATR FTIR (diamond crystal) (Bruker, Billerica, MA, USA) spectrometer using potassium bromide (KBr) pellets, operating range 400-4000 cm^{-1}.

The ^{1}H and ^{13}C NMR spectra of the samples were recorded using a JNM-ECA 400 (JEOL, Tokyo, Japan) spectrometer, operating at frequencies of 399.78 MHz for ^{1}H and 100.53 MHz for ^{13}C, in deuterated dimethyl sulfoxide (DMSO-d_6). The residual signal of solvent DMSO-d_6 at 2.5 ppm was used as a chemical shift standard.

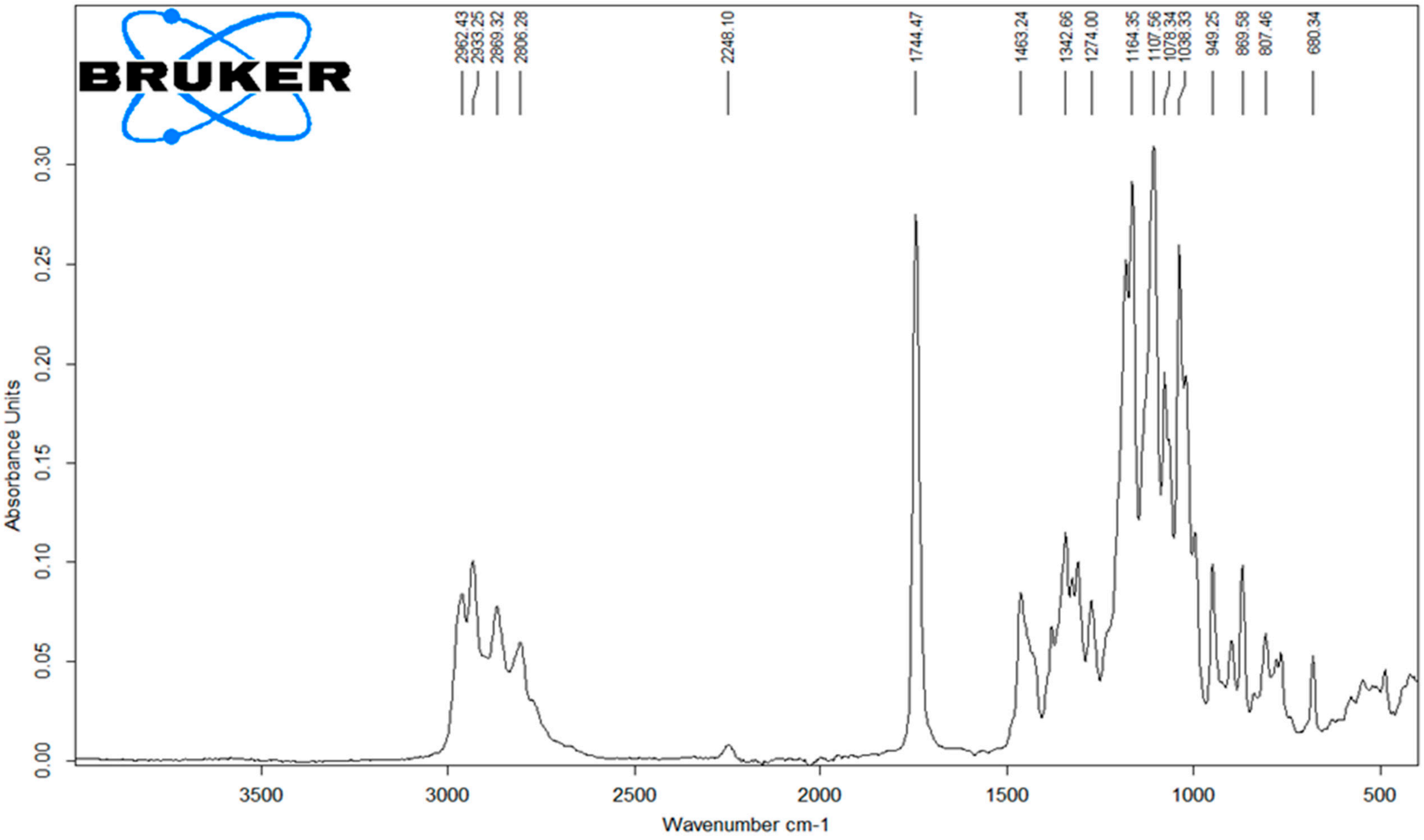

Figure S1. IR (KBr, ν, cm^{-1}) spectrum of 1-(2-ethoxyethyl)-4-(pent-1-yn-1-yl)piperidinyl-4 propionate (***EPPP***).

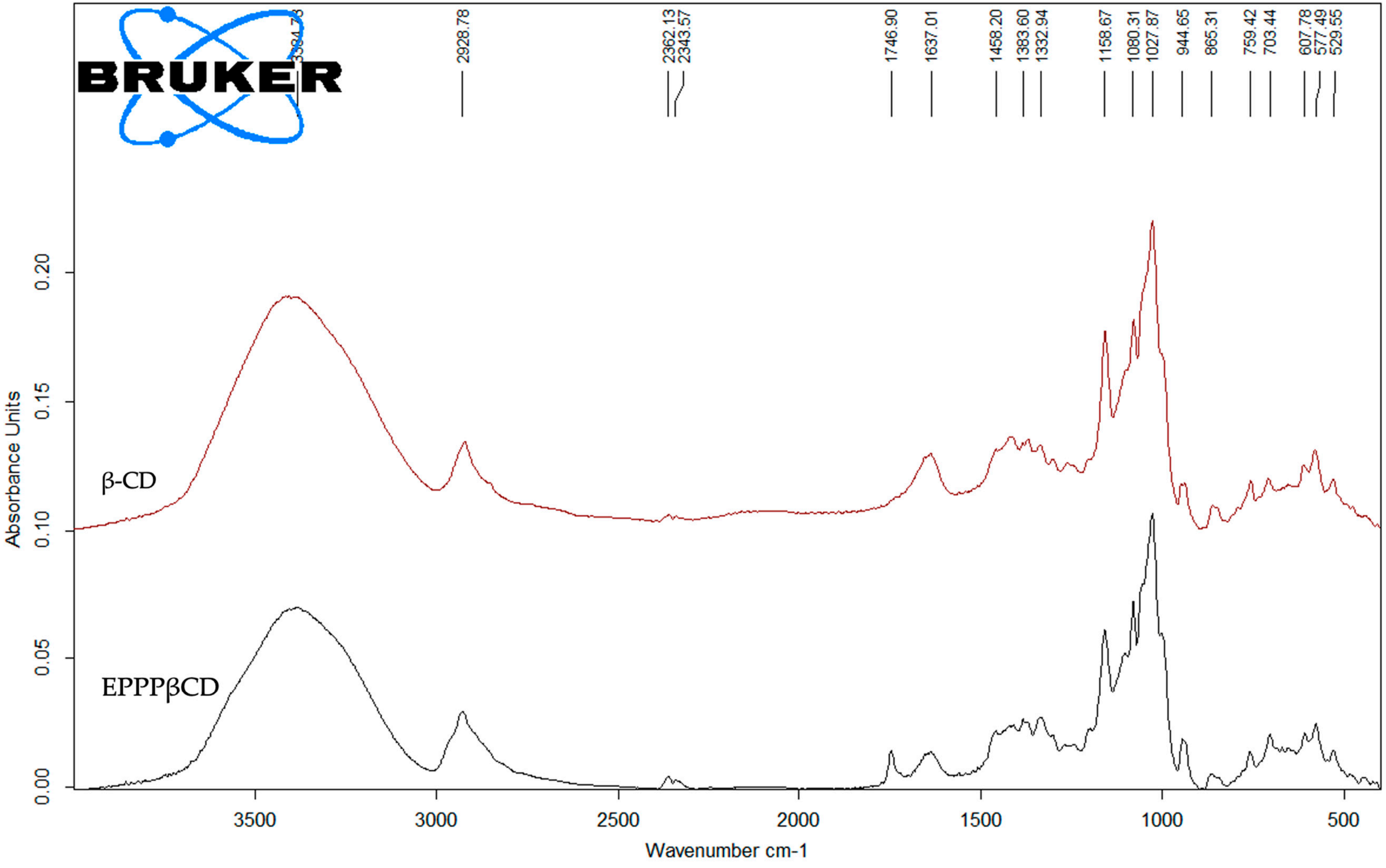

Figure S2. IR (KBr, ν, cm^{-1}) spectrum of β-cyclodextrin (β-CD) and the complex of 1-(2-ethoxyethyl)-4-(pent-1-yn-1-yl)piperidin-4-yl propionate with β-cyclodextrin (***EPPPβCD***).

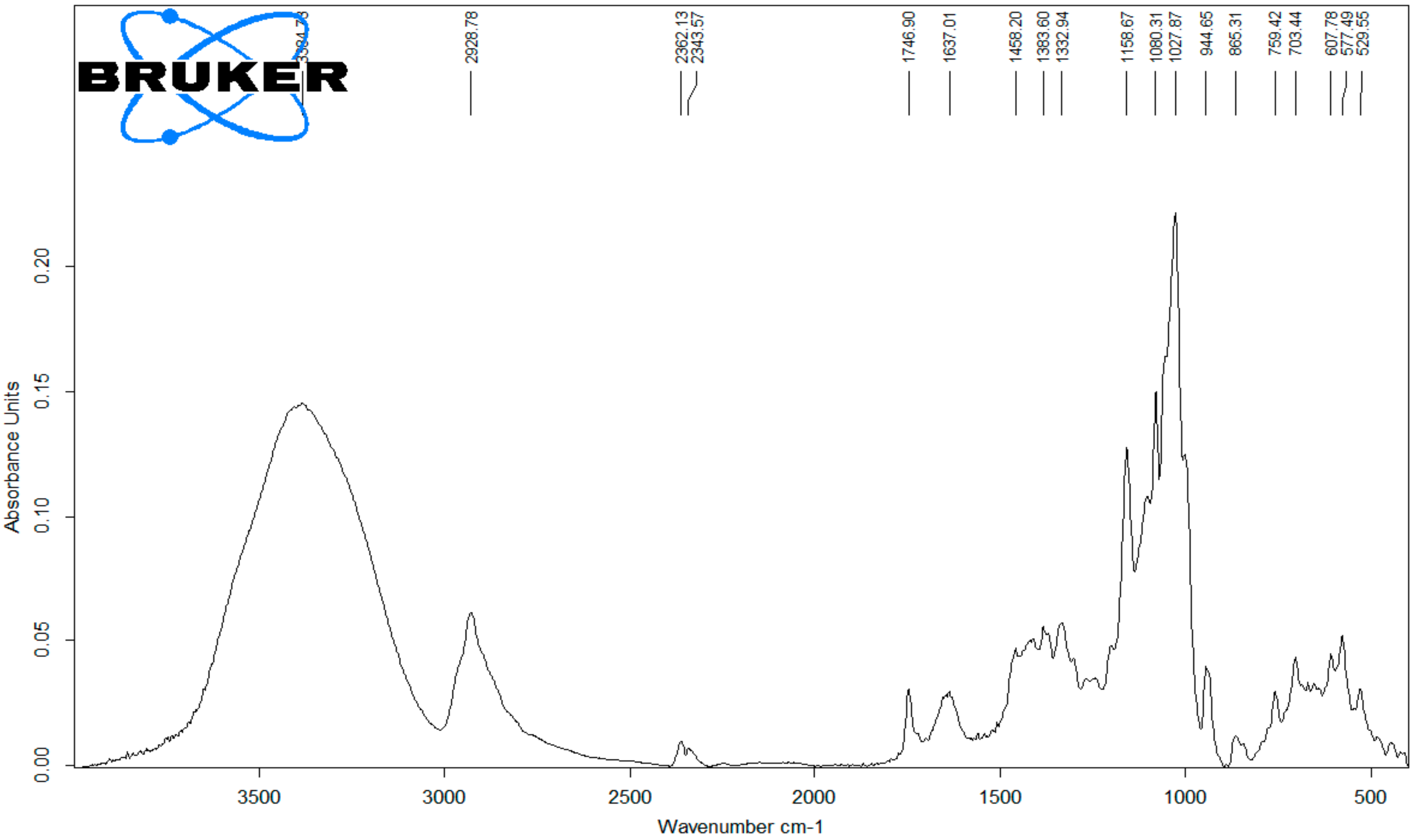

Figure S3. IR (KBr, ν, cm^{-1}) spectrum of the complex of 1-(2-ethoxyethyl)-4-(pent-1-yn-1-yl)piperidin-4-yl propionate with β-cyclodextrin (***EPPPβCD***).

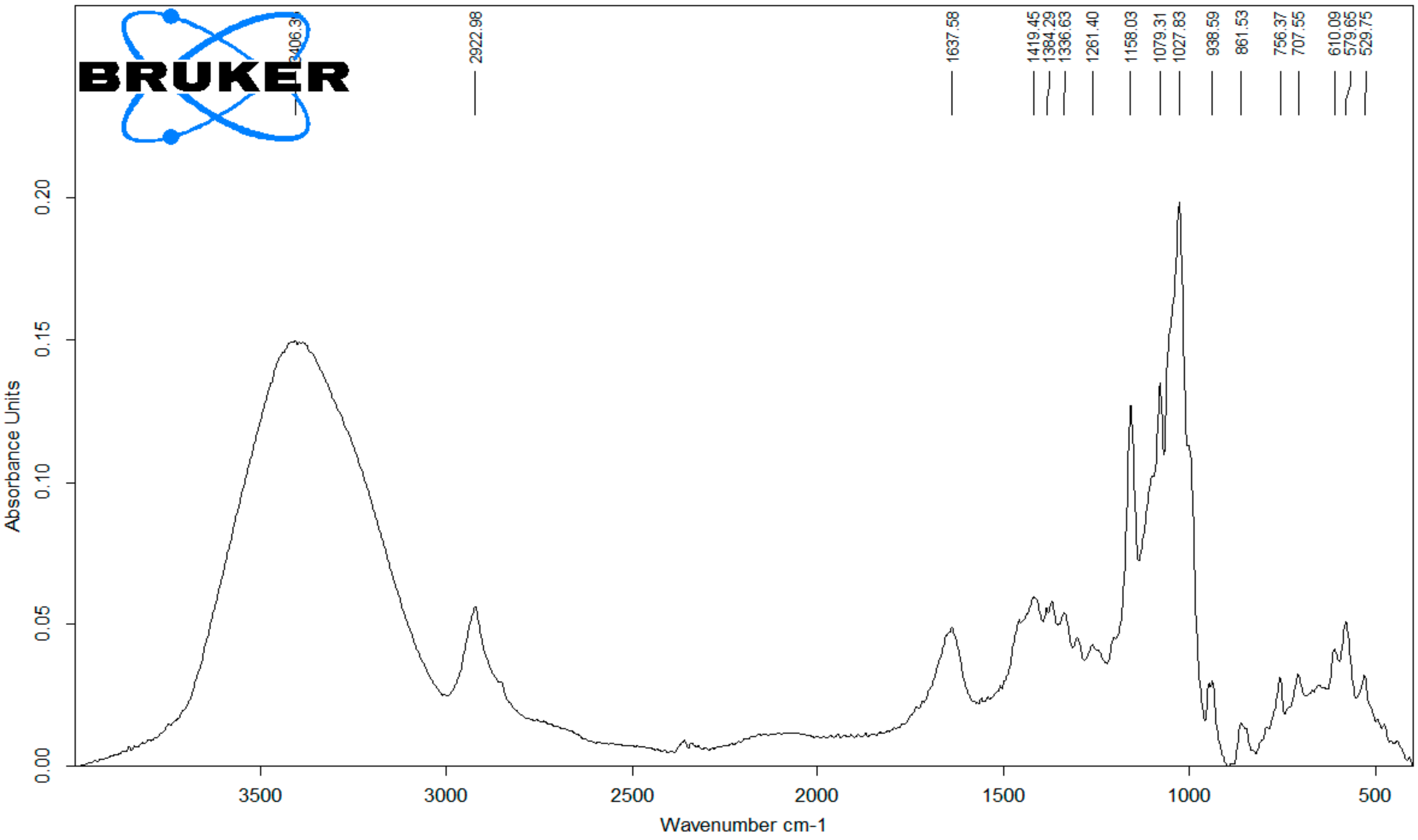

Figure S4. IR (KBr, ν, cm^{-1}) spectrum of β-cyclodextrin (β-CD).

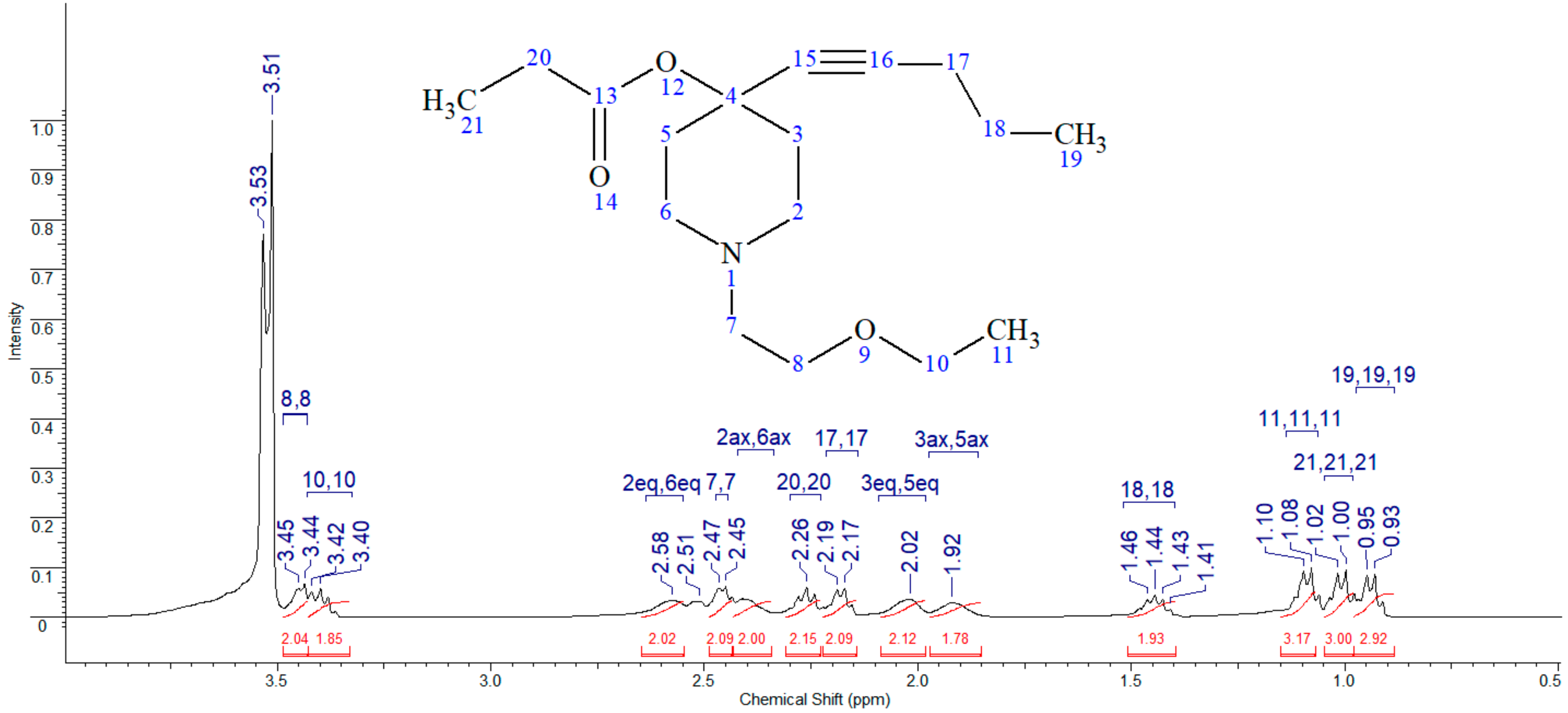

Figure S5. ^{1}H NMR (399.78 MHz, DMSO-d6) spectrum of 1-(2-ethoxyethyl)-4-(pent-1-yn-1-yl)piperidinyl-4 propionate (***EPPP***).

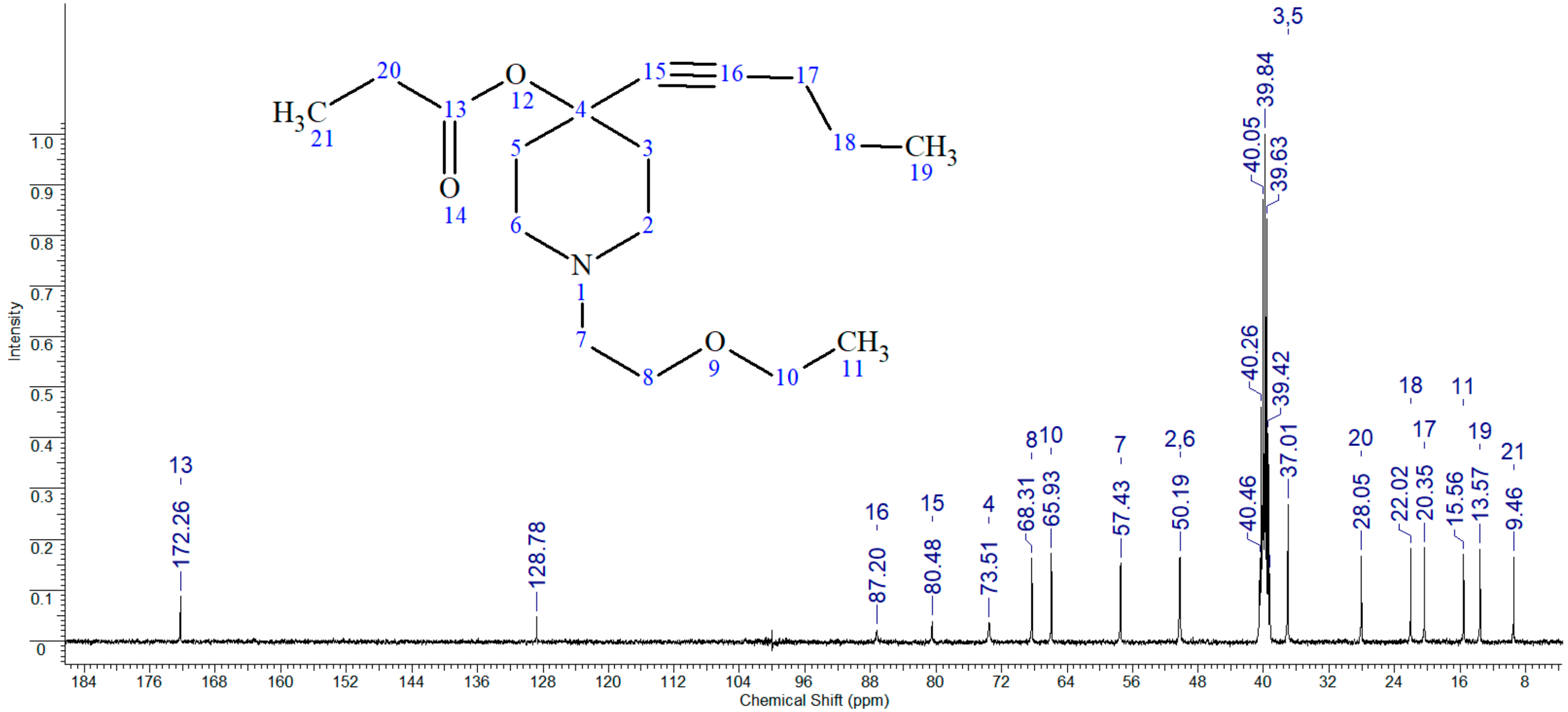

Figure S6. ^{13}C NMR (100.53 MHz, DMSO-d$_6$) spectrum of 1-(2-ethoxyethyl)-4-(pent-1-yn-1-yl)piperidinyl-4 propionate (***EPPP***).

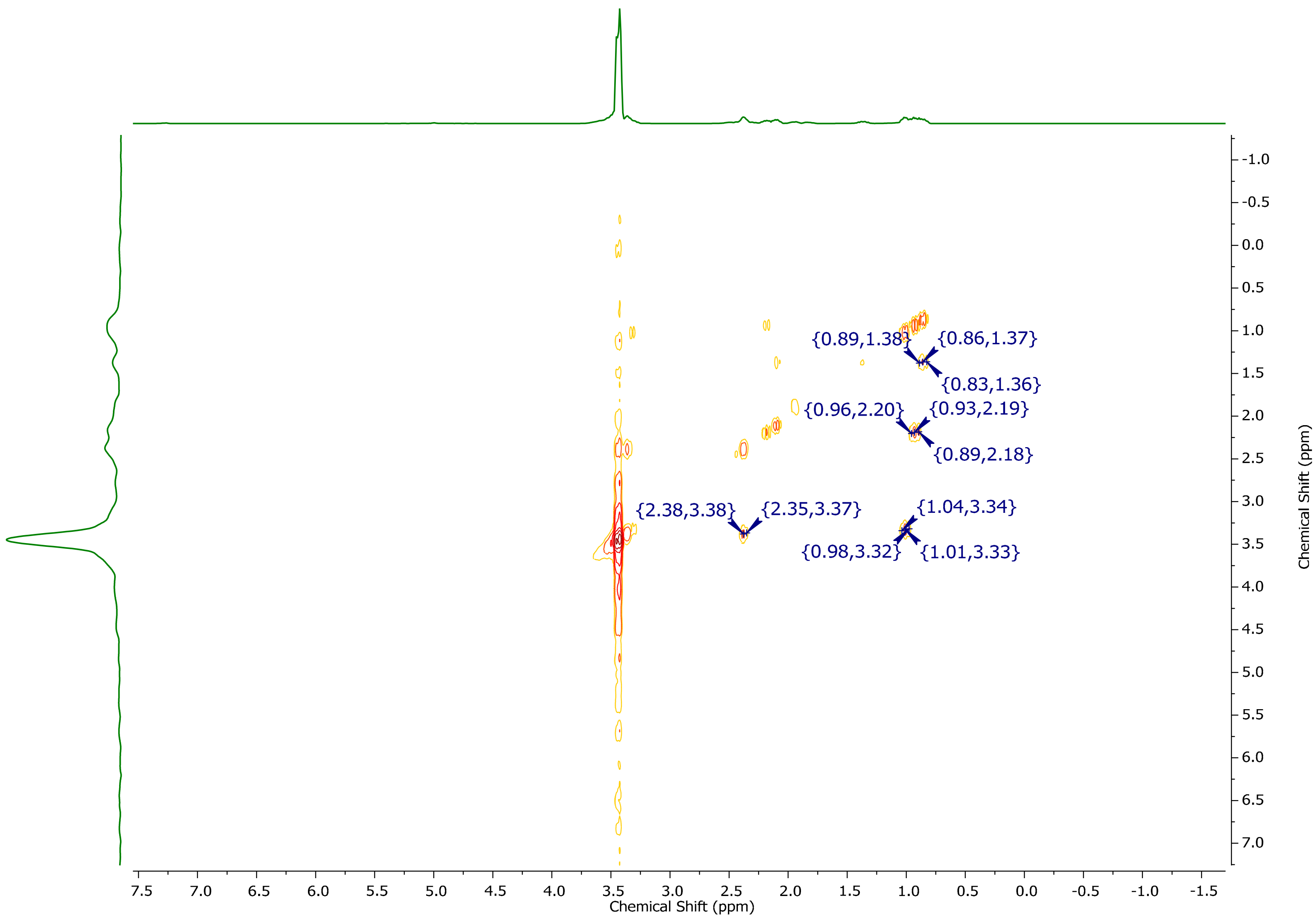

Figure S7. ^{1}H-^{1}H COSY spectrum of 1-(2-ethoxyethyl)-4-(pent-1-yn-1-yl)piperidinyl-4 propionate (***EPPP***).

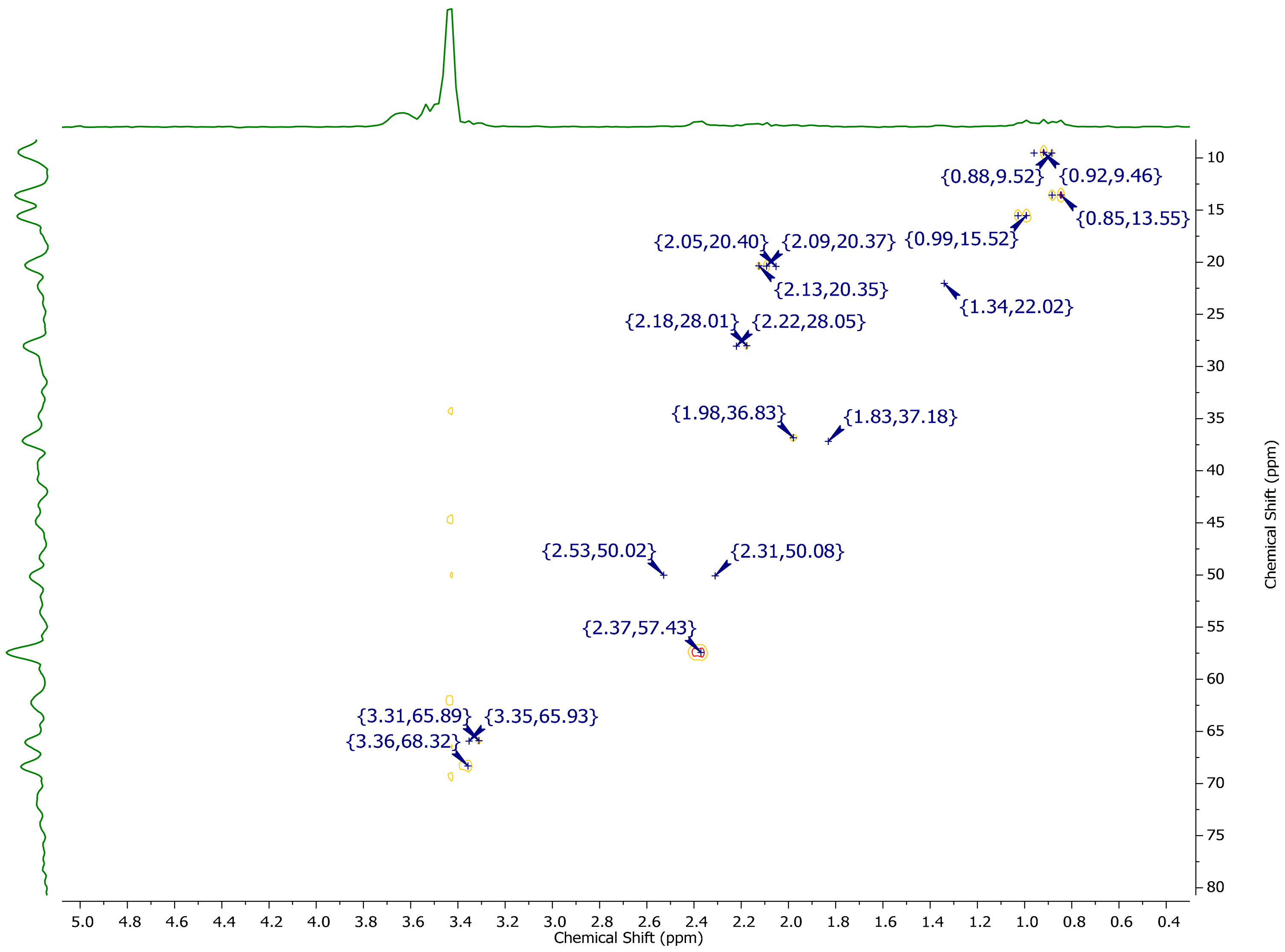

Figure S8. ^{1}H-^{13}C HMQC spectrum of 1-(2-ethoxyethyl)-4-(pent-1-yn-1-yl)piperidinyl-4 propionate (***EPPP***).

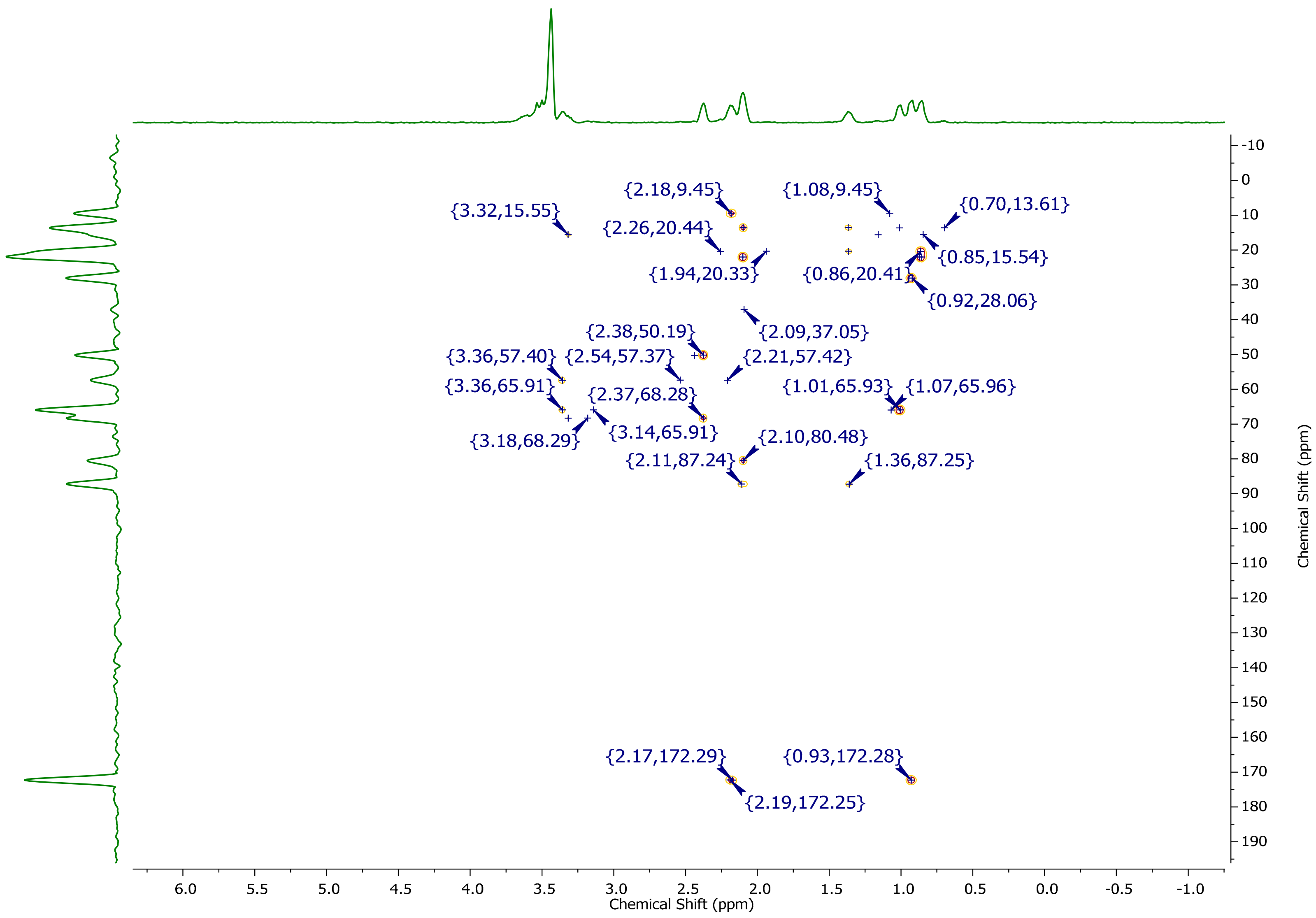

Figure S9. ^{1}H-^{13}C HMBC spectrum of 1-(2-ethoxyethyl)-4-(pent-1-yn-1-yl)piperidinyl-4 propionate (***EPPP***).

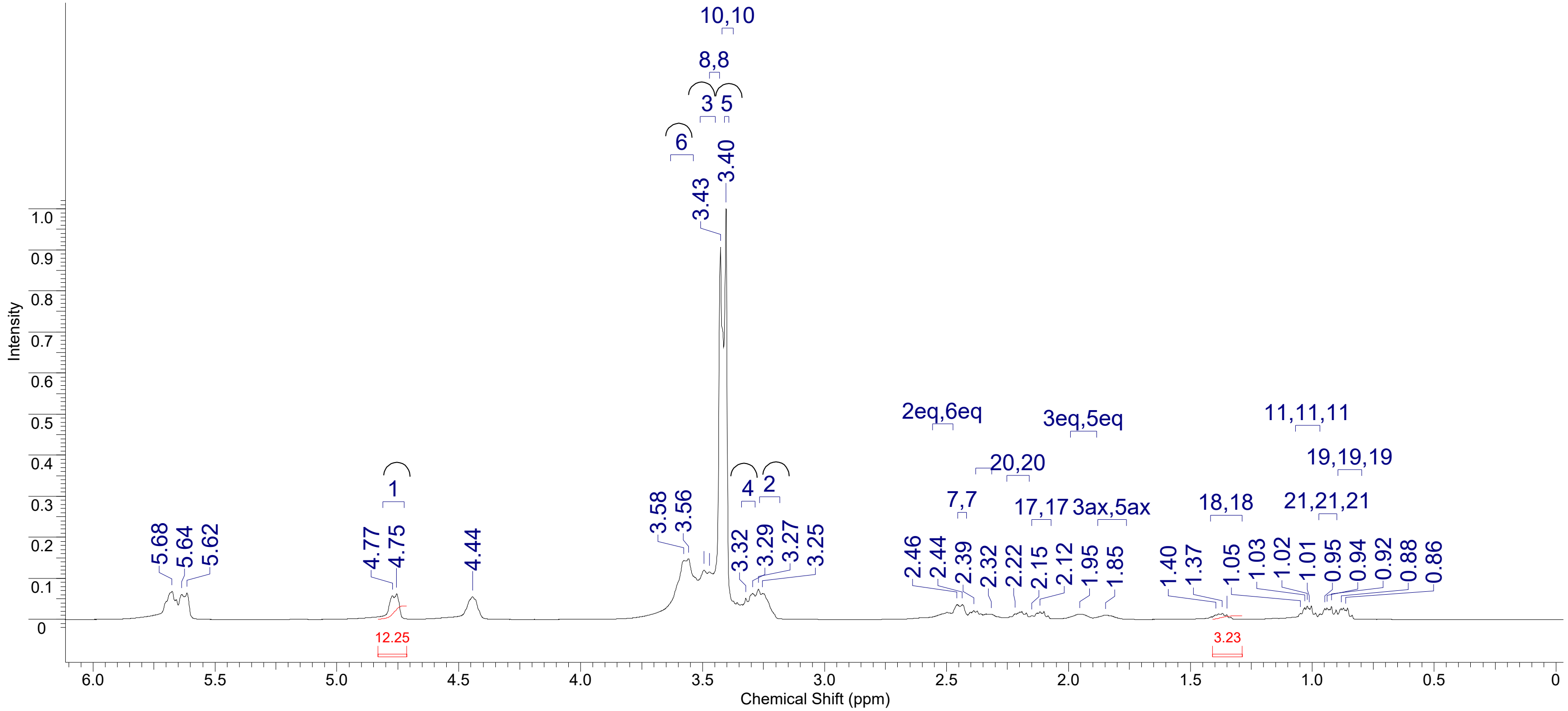

Figure S10. ^{1}H NMR (399.78 MHz, DMSO-d$_6$) spectrum of the complex of 1-(2-ethoxyethyl)-4-(pent-1-yn-1-yl)piperidin-4-yl propionate with β-cyclodextrin (***EPPPβCD***).

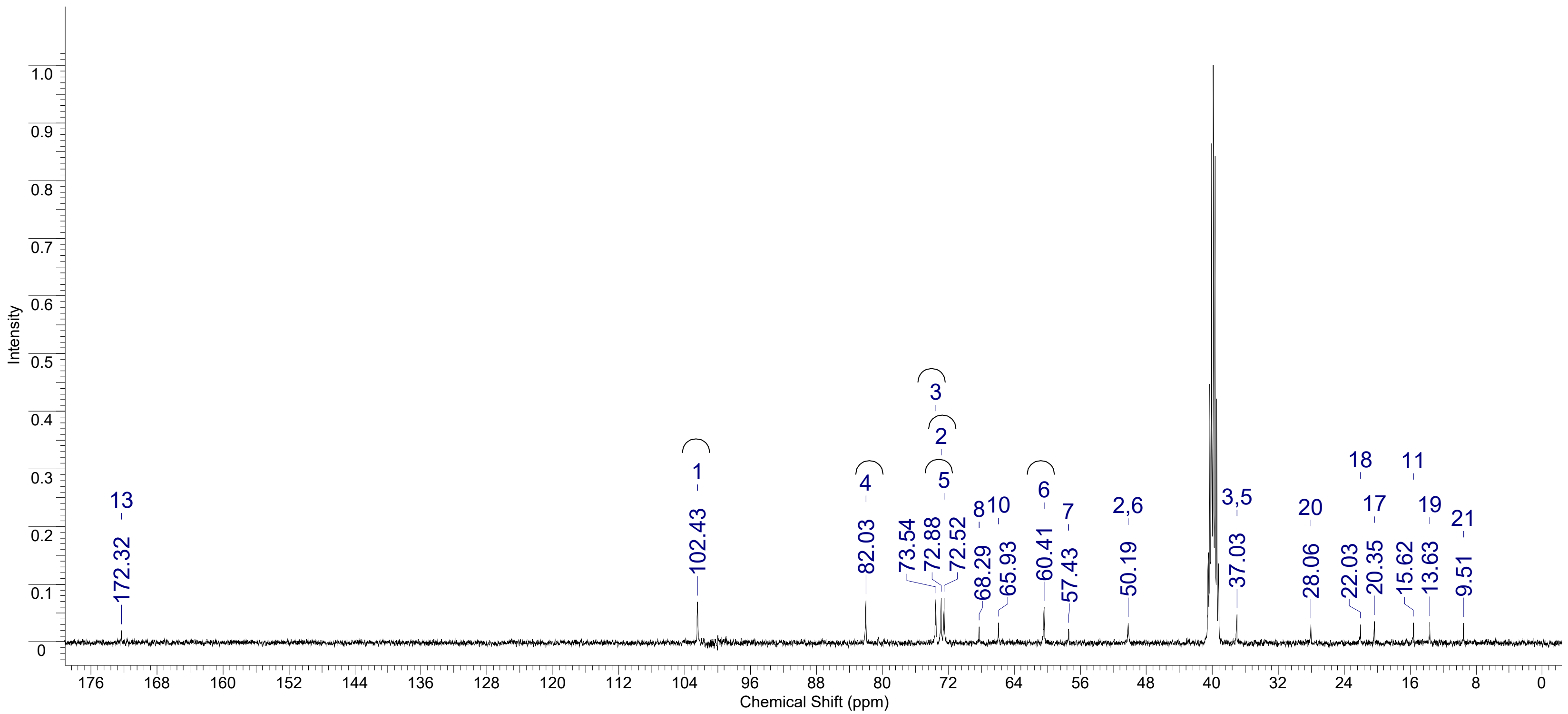

Figure S11. ^{13}C NMR (100.53 MHz, DMSO-d$_6$) spectrum of the complex of 1-(2-ethoxyethyl)-4-(pent-1-yn-1-yl)piperidin-4-yl propionate with β-cyclodextrin (***EPPPβCD***).

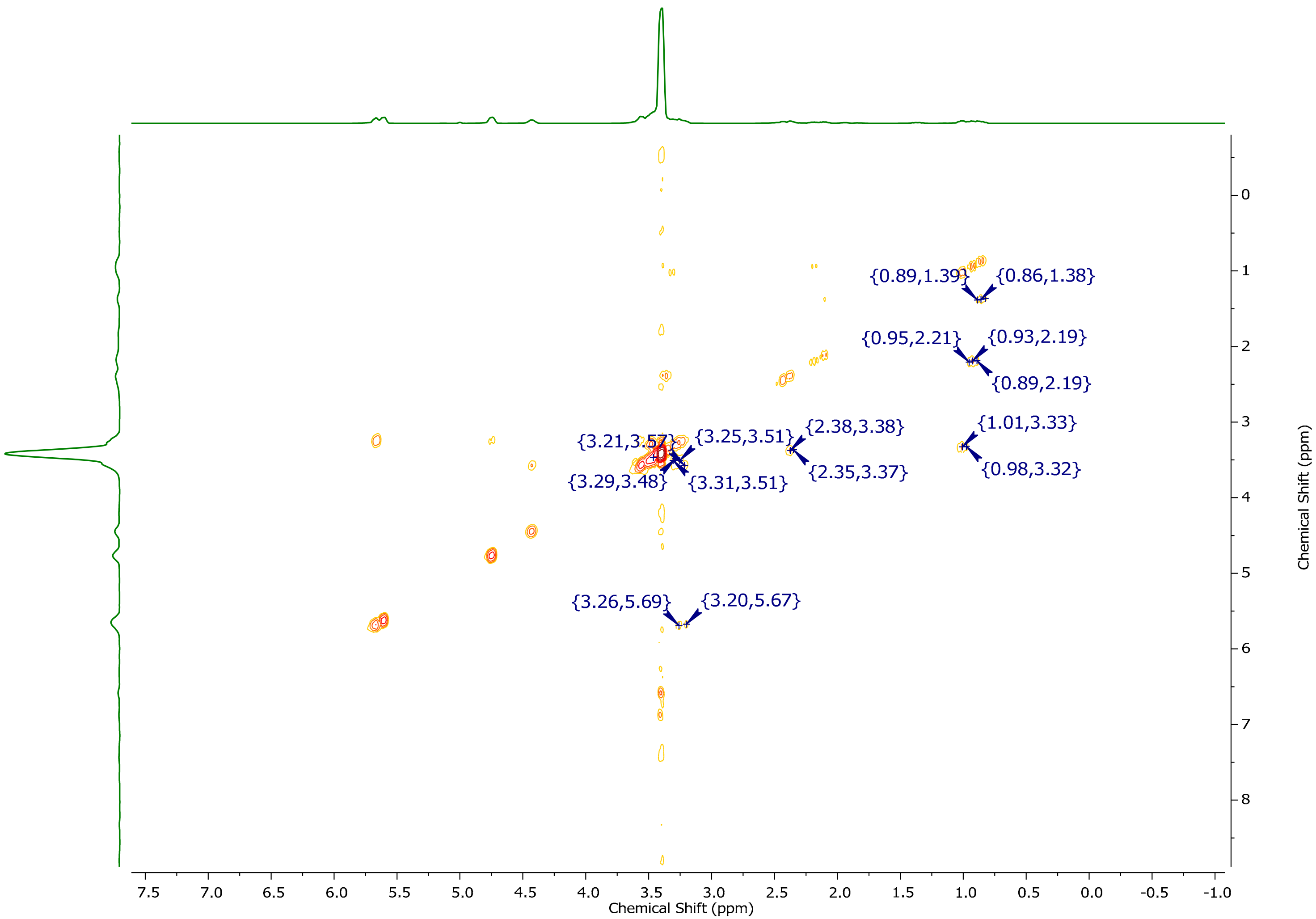

Figure S12. ^{1}H-^{1}H COSY spectrum of the complex of 1-(2-ethoxyethyl)-4-(pent-1-yn-1-yl)piperidin-4-yl propionate with β-cyclodextrin (***EPPPβCD***).

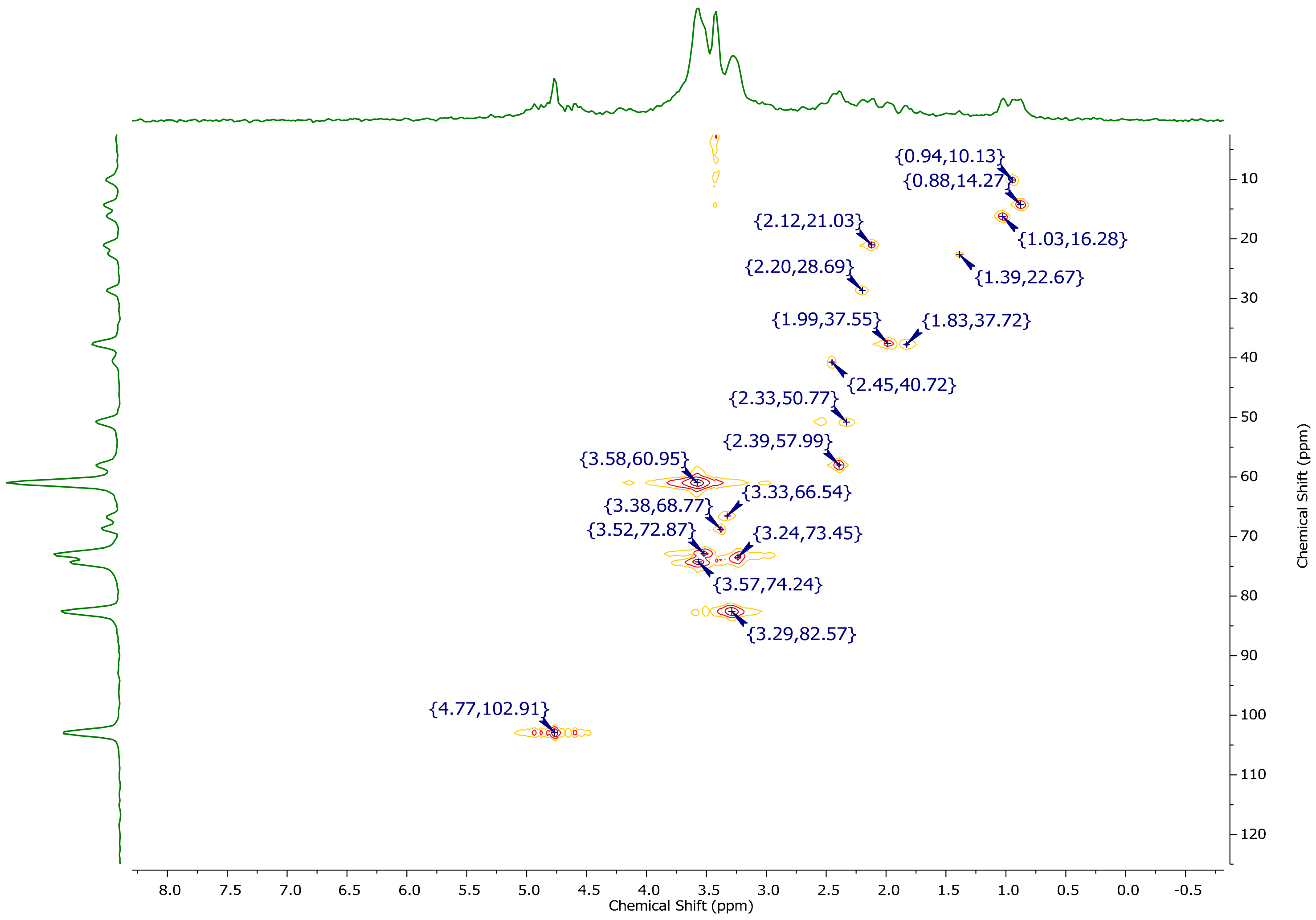

Figure S13. ^{1}H-^{13}C HMQC spectrum of the complex of 1-(2-ethoxyethyl)-4-(pent-1-yn-1-yl)piperidin-4-yl propionate with β-cyclodextrin (***EPPPβCD***).

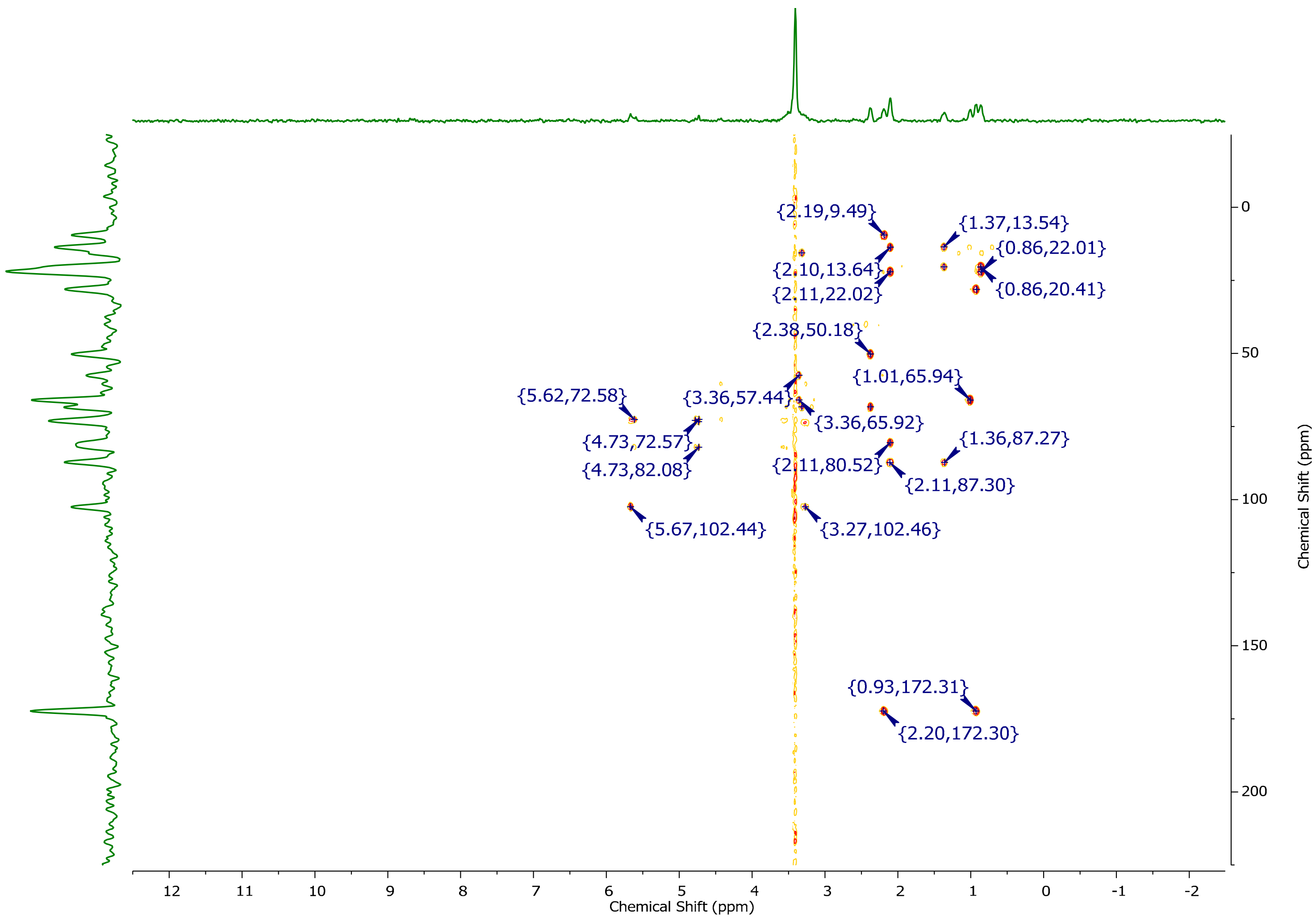

Figure S14. ^{1}H-^{13}C HMBC spectrum of the complex of 1-(2-ethoxyethyl)-4-(pent-1-yn-1-yl)piperidin-4-yl propionate with β-cyclodextrin (***EPPPβCD***).

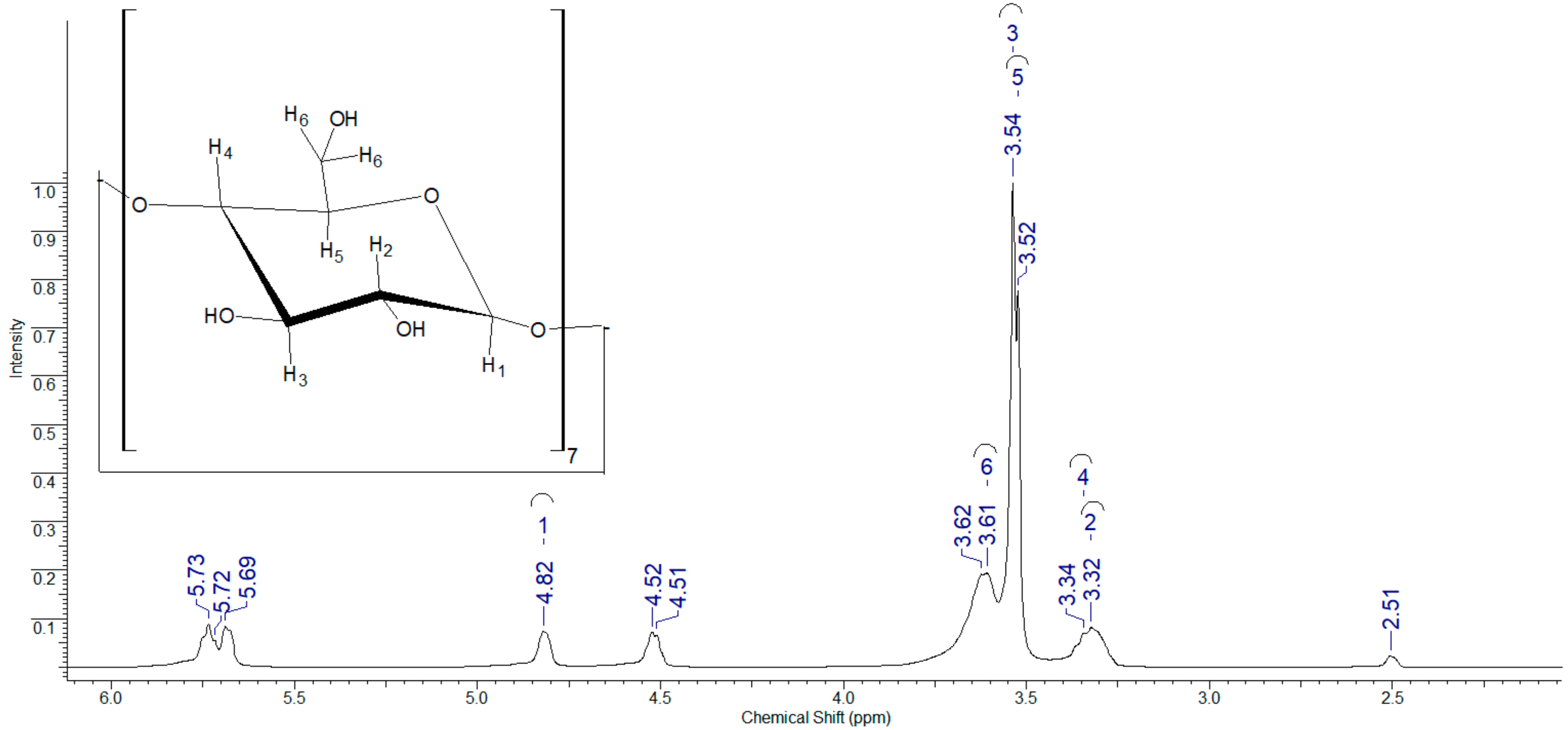

Figure S15. ^{1}H NMR (399.78 MHz, DMSO-d$_6$) spectrum of β-cyclodextrin (β-CD).

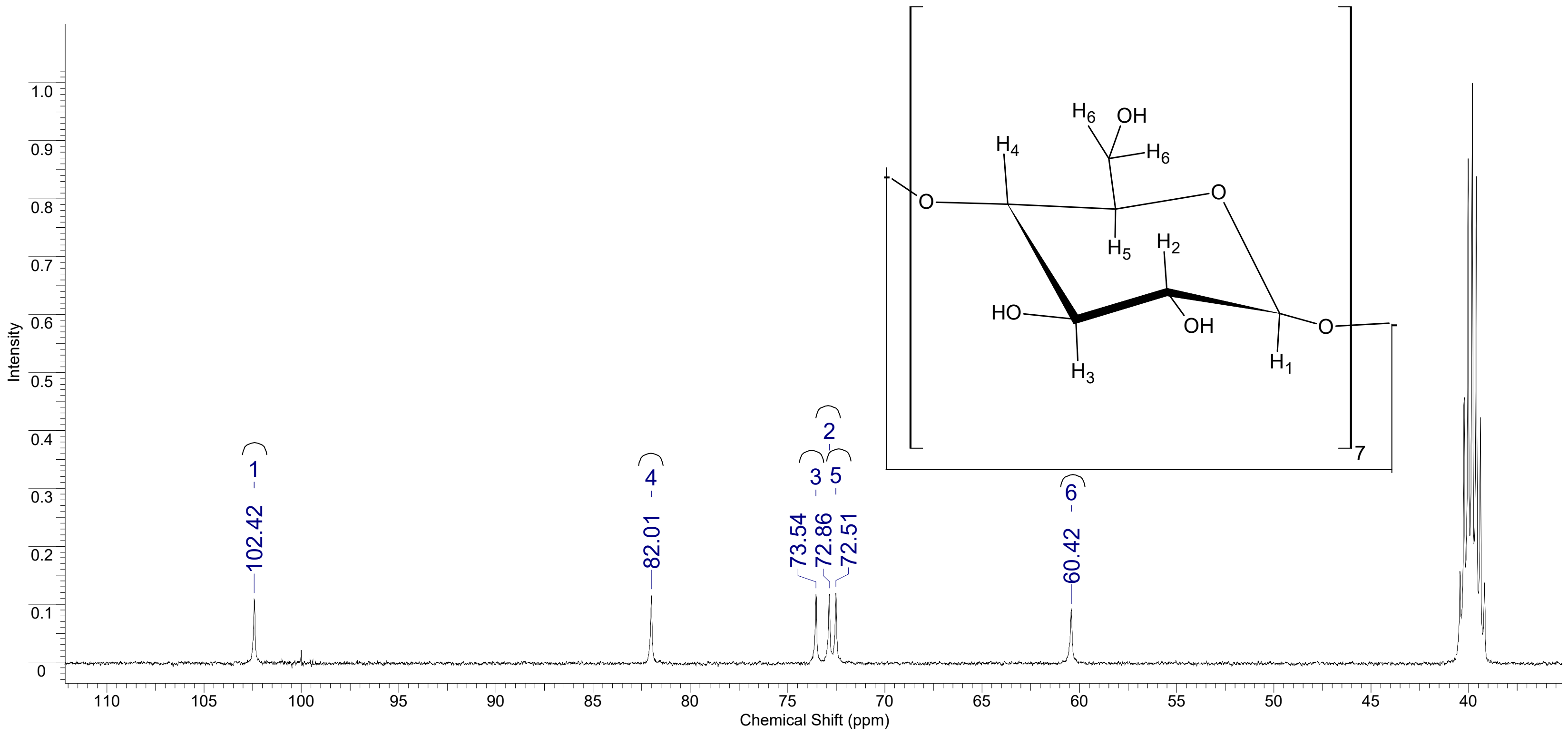

Figure S16. ^{13}C NMR (100.53 MHz, DMSO-d$_6$) spectrum of β-cyclodextrin (β-CD).

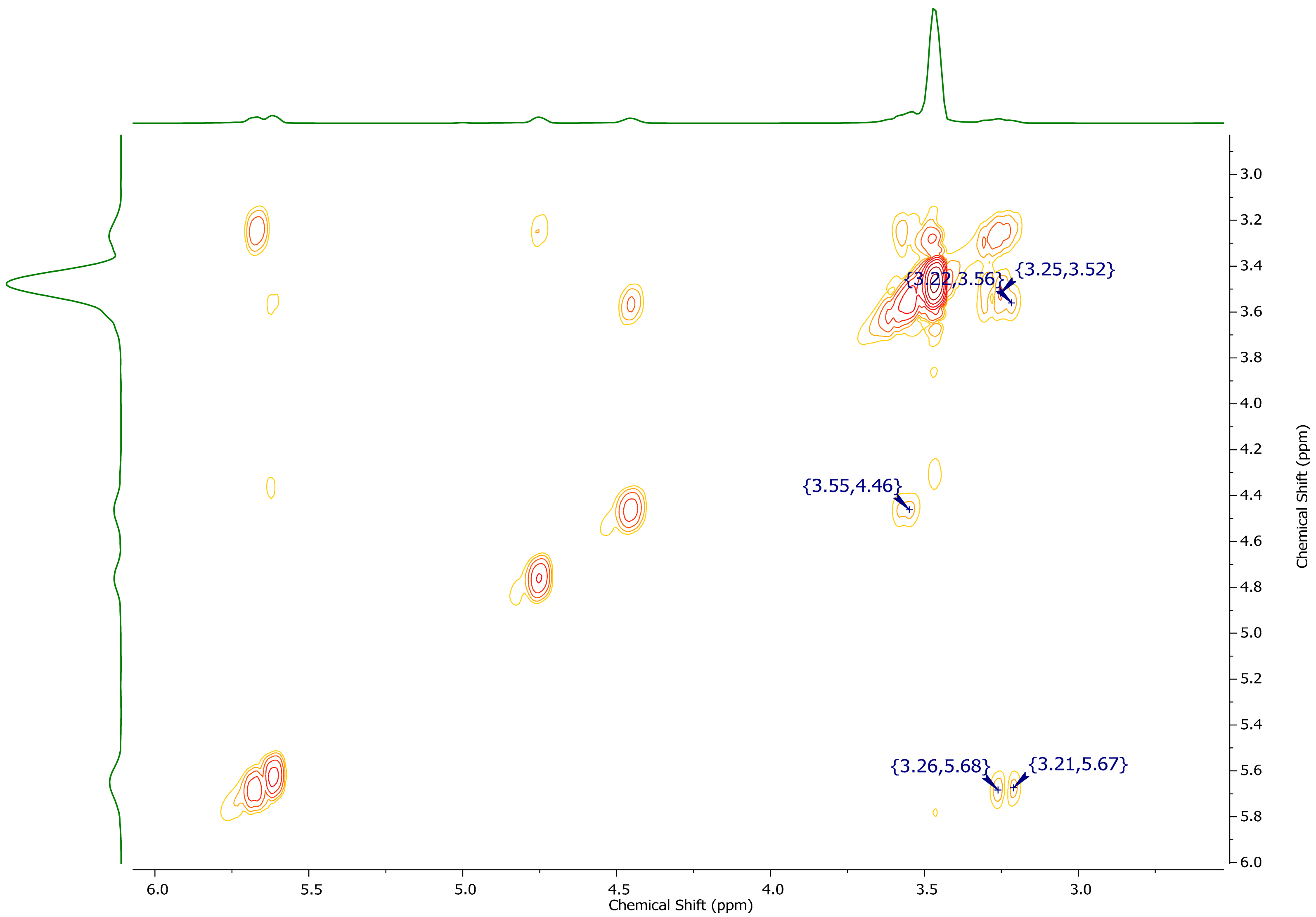

Figure S17. ^{1}H-^{1}H COSY spectrum of β-cyclodextrin (β-CD).

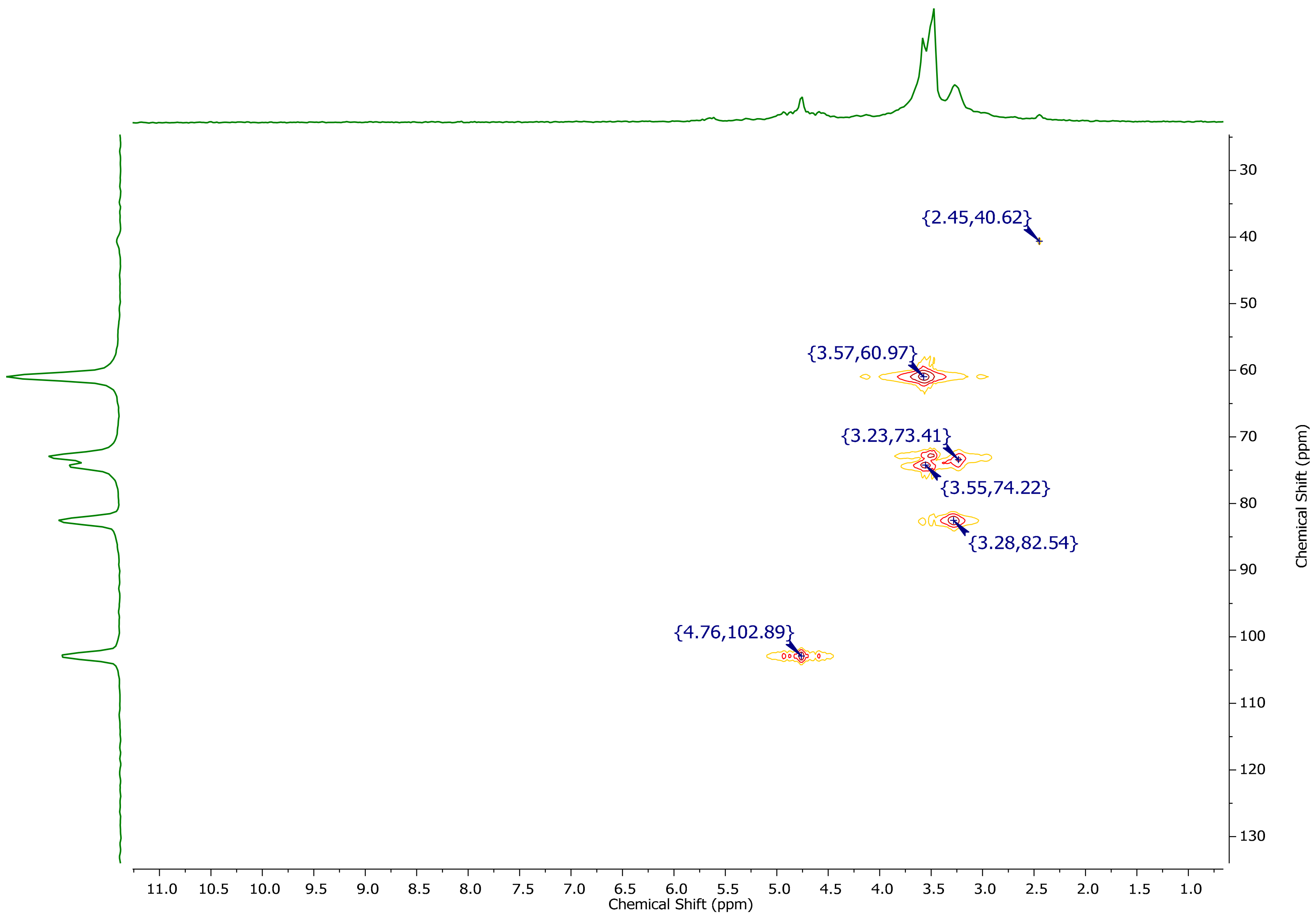

Figure S18. ^{1}H-^{13}C HMQC spectrum of β-cyclodextrin (β-CD).

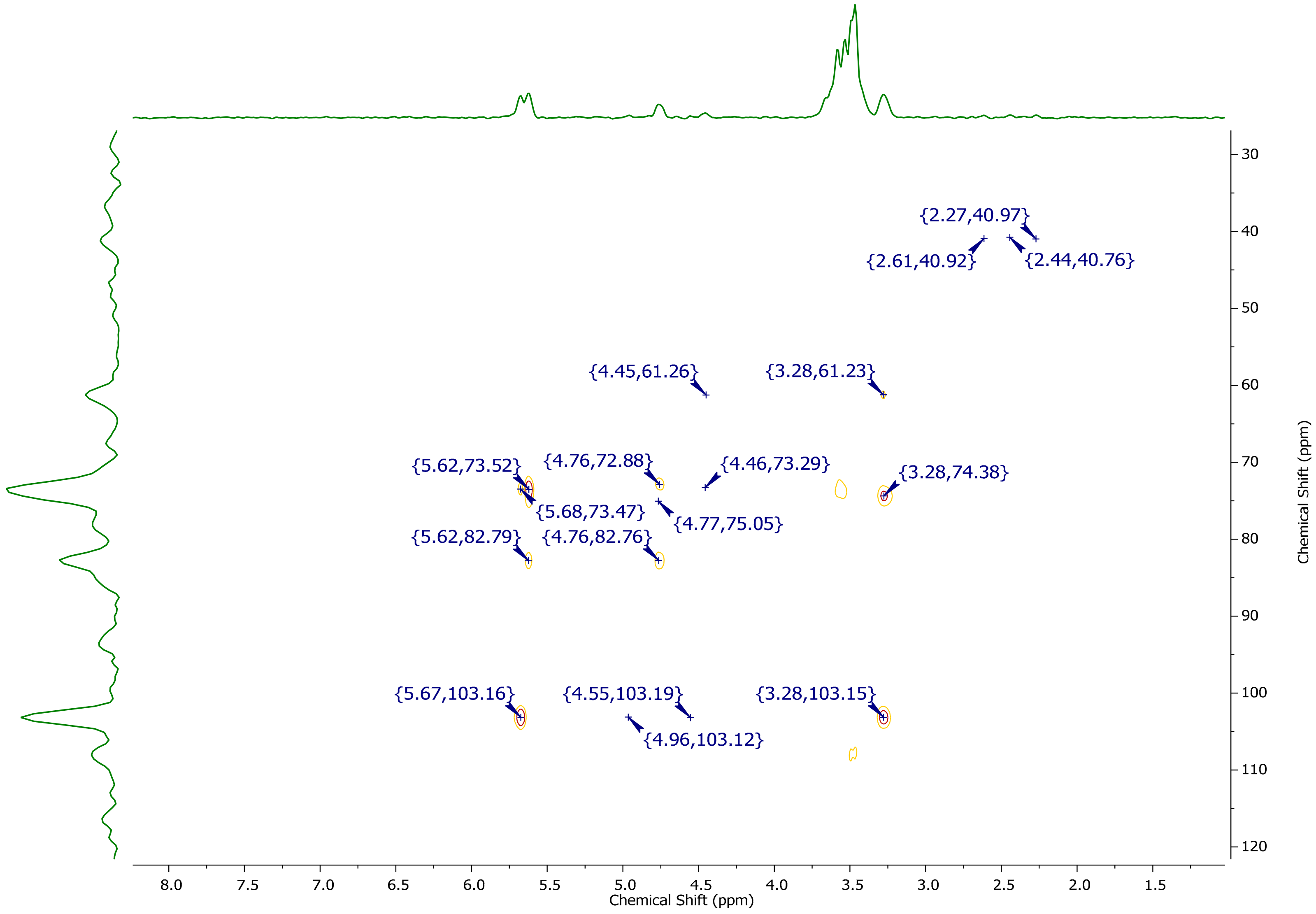

Figure S19. ^{1}H-^{13}C HMBC spectrum of β-cyclodextrin (β-CD).
